# Adaptation of *Candida albicans* to environmental pH induces cell wall remodelling and enhances innate immune recognition

**DOI:** 10.1371/journal.ppat.1006403

**Published:** 2017-05-22

**Authors:** Sarah L. Sherrington, Eleanor Sorsby, Nabeel Mahtey, Pizga Kumwenda, Megan D. Lenardon, Ian Brown, Elizabeth R. Ballou, Donna M. MacCallum, Rebecca A. Hall

**Affiliations:** 1 Institute of Microbiology and Infection, and School of Biosciences, University of Birmingham, Edgbaston, Birmingham, United Kingdom; 2 MRC Centre for Medical Mycology, Aberdeen Fungal Group, Institute of Medical Sciences, University of Aberdeen, Aberdeen, United Kingdom; 3 School of Biosciences, University of Kent, Canterbury, Kent, United Kingdom; Louisiana State University Health Sciences Center New Orleans, UNITED STATES

## Abstract

*Candida albicans* is able to proliferate in environments that vary dramatically in ambient pH, a trait required for colonising niches such as the stomach, vaginal mucosal and the GI tract. Here we show that growth in acidic environments involves cell wall remodelling which results in enhanced chitin and β-glucan exposure at the cell wall periphery. Unmasking of the underlying immuno-stimulatory β-glucan in acidic environments enhanced innate immune recognition of *C*. *albicans* by macrophages and neutrophils, and induced a stronger proinflammatory cytokine response, driven through the C-type lectin-like receptor, Dectin-1. This enhanced inflammatory response resulted in significant recruitment of neutrophils in an intraperitoneal model of infection, a hallmark of symptomatic vaginal colonisation. Enhanced chitin exposure resulted from reduced expression of the cell wall chitinase Cht2, via a Bcr1-Rim101 dependent signalling cascade, while increased β-glucan exposure was regulated via a non-canonical signalling pathway. We propose that this “unmasking” of the cell wall may induce non-protective hyper activation of the immune system during growth in acidic niches, and may attribute to symptomatic vaginal infection.

## Author summary

To be able to colonise a host or cause infection, microbes must be able to adapt and respond to changes in their environment. One of the most important environmental signals for opportunistic pathogens is ambient pH, with changes in external pH resulting in phenotypic, metabolic and physical changes in fungi and bacteria (i.e. *E*. *coli*, *Salmonella*, *Aspergillus*, *Candida*). *Candida albicans* is an opportunistic fungal pathogen of humans that can colonise and infect niches of varying pH including the acidic mucosa of the vagina. Here we show that growth in an acidic environment results in structural modification of the fungal cell wall, a dynamic organelle key to immune recognition. These cell wall perturbations resulted in enhanced immune recognition of the fungal pathogen, a strong proinflammatory immune response and enhanced recruitment of neutrophils. Therefore, colonisation of acidic mucosa may result in the unmasking of cell wall components that trigger hyper-activation of the innate immune response and could contribute to immunopathology associated with vaginal candidiasis.

## Introduction

The opportunistic fungal pathogen *Candida albicans* is a commensal in up to 80% of the population, and can cause superficial mucosal infections in healthy individuals [1, 2] and invasive disease in immune supressed patients [3, 4]. Mucosal infections increase population morbidity and are expensive to treat, while disseminated disease is associated with high mortality rates [5, 6].

One attribute of *C*. *albicans* that has made it such a successful opportunistic pathogen is its ability to adapt and proliferate in a broad range of host environments. One of the most important environmental conditions that fluctuate between different niches is ambient pH. *C*. *albicans* is able to grow in media ranging from pH2 to pH10, and *C*. *albicans* has been isolated from a range of anatomical sites that vary dramatically in ambient pH including the stomach (pH2) [7], vagina (pH4-5) [8] and the oral mucosa (pH6) [9], suggesting that adaptation to environmental pH is key to the pathogenicity of *C*. *albicans*. Adaptation of *C*. *albicans* to acidic environments regulates key biological processes including morphogenesis [10], white-to-opaque switching, and mating [11]. However, the impact environmental adaptation has on the structure and composition of the fungal cell wall, the first point of contact between the fungus and host, is not well defined.

The fungal cell wall is a complex, multi-layered structure of mannoproteins, β-glucans and chitin that provides rigidity and shape and protects the fungus from the environment [12]. These protein and carbohydrate motifs are immunogenic and play important roles in innate immune recognition [12]. Environmental adaptation influences the cell wall proteome and impacts on the structure of the glycans that decorate the cell wall proteins [13]. For instance, growth in blood or lactate media decreases the structural complexity of the outer mannan fibrils [14–16], while antifungal drug treatment influences β-glucan exposure [17, 18]. Structural changes in the cell wall, as a result of mutation in key glycolytic cell wall assembly enzymes, confirm that modulation of the cell wall has profound implications for innate immune recognition [19]. Therefore, understanding how environmental adaptation impacts on cell wall biogenesis, and the consequence this cell wall remodelling has on innate immune recognition is an important, but understudied, area of fungal biology. Here, we investigate how adaptation to acidic environments that mimic the pH of the vaginal mucosa impact on the structure and composition of the *C*. *albicans* cell wall and deduce how this cell wall remodelling affects the innate immune recognition of the pathogen.

## Results

### Growth in acidic environments alters the ultrastructure of the fungal cell wall

**Fig 1:**
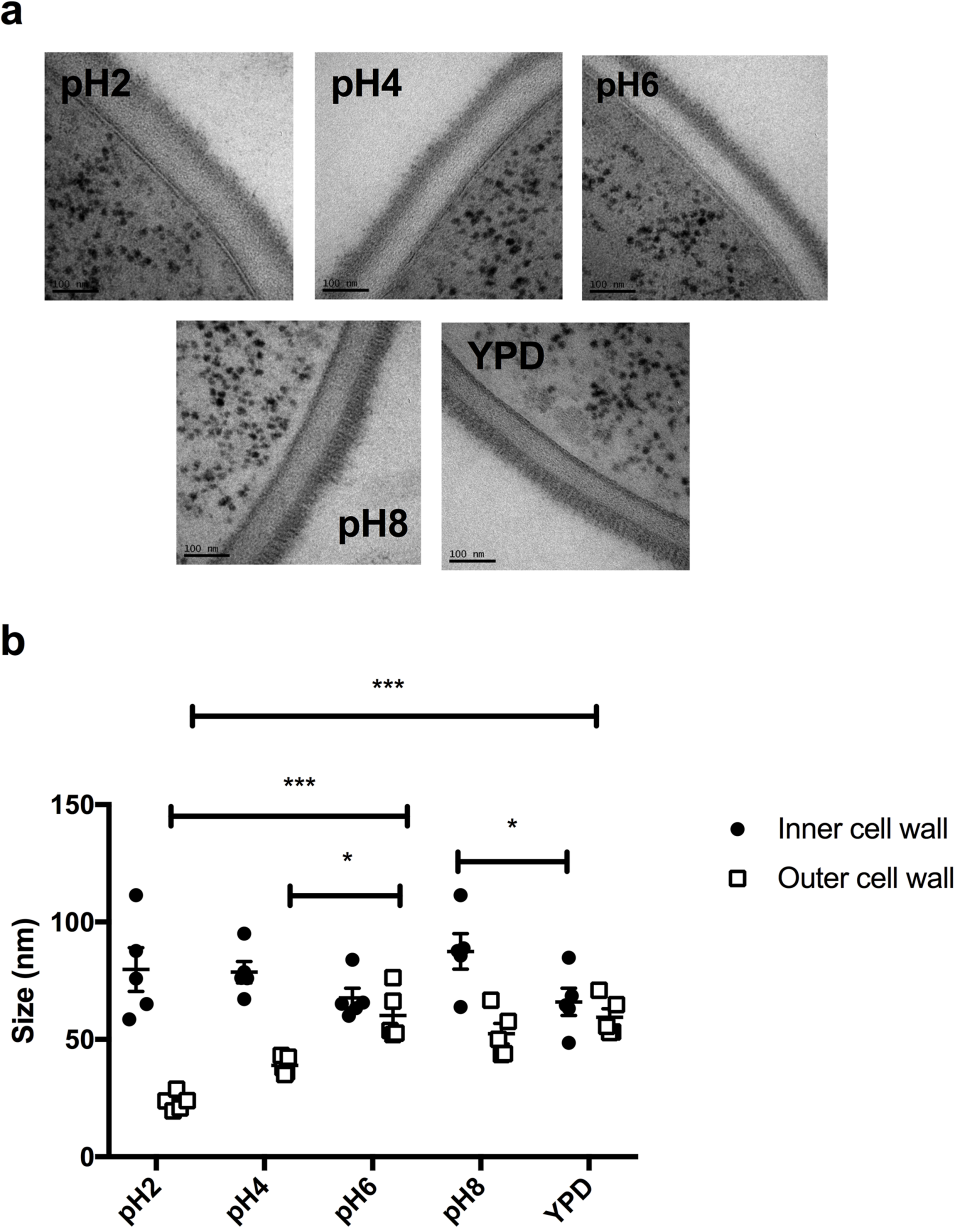
Adaptation to environmental pH alters the ultrastructure of the fungal cell wall. **a)** Electron micrographs showing the ultrastructure of the cell walls of wild type *C*. *albicans* (NGY152) grown to mid-log phase in YPD buffered to the appropriate pH. Scale bar represents 100 nm. **b)** Quantification of the thickness of the inner cell wall layer and mannoprotein fibril length. Data represent the mean ± SEM from 5 individual cells. Each cell was measured at 30 different points around the cell periphery (* p < 0.05, ** p < 0.01, *** p < 0.001).

The impact of adaptation to environmental pH on the ultrastructure of the cell wall was investigated via transmission electron microscopy. The cell wall of mid-log phase yeast cells grown in YPD buffered at pH2, pH4 pH6, pH8 and standard YPD were imaged. Under all tested conditions, the cell wall maintained two distinct layers: an inner layer comprised of glucan and chitin, and an outer, fibrillar layer of mannoproteins. However, adaptation to acidic conditions resulted in a significant loss of structural organisation in the outer cell wall, which appeared to be less fibrillar (Fig 1A). Quantification of the thickness of the outer cell wall layer confirmed that adaptation to acidic environments significantly reduced mannan fibril length (pH2 23.23 ± 3.58 nm (p = 0.0001), pH4 39.49 ± 4.61 nm (p = 0.0495), compared to pH6 60.22 ± 10.73 nm; Fig 1B). We hypothesized that this loss of fibrillar structure might be due to changes in the underlying architecture of the cell wall. Therefore, we investigated whether pH similarly influences chitin structure.

### Acidic environments induce reorganisation of the fungal cell wall through modulation of chitin

**Fig 2:**
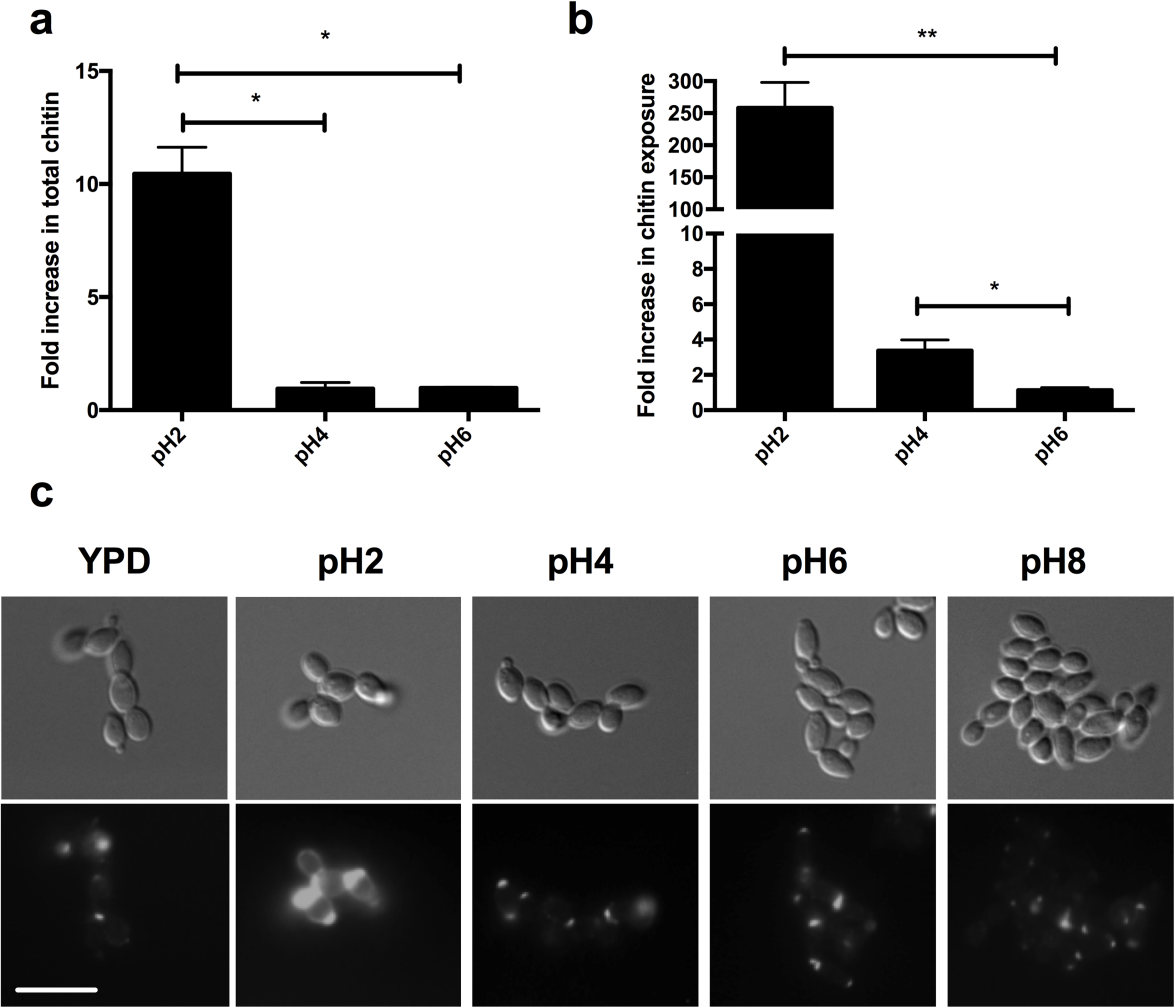
Adaptation to acidic environments promotes surface exposure of chitin. **a)** Wild type cells (NYG152) were grown in YPD buffered at the appropriate pH, stained with CFW and fluorescence quantified by FACS. The fold increase in CFW staining was determined from the background subtracted MFI values from FACS analysis, and normalised to YPD grown cells. **b)** Fold increase in FITC fluorescence of FITC-WGA stained wild type cells (NYG152) grown at different pH, relative to growth in YPD as quantified by FACS analysis. All data represent the mean ± SEM from three independent experiments. **c)** Microscopy of WGA stained cells. Arrowheads indicate chitin exposure. Scale bar = 10 μm.

Although chitin only forms a small fraction of the fungal cell wall (3–5% by dry weight), a major compensatory mechanism of *C*. *albicans* to cell wall stress is to up-regulate chitin synthesis to provide increased cell wall integrity [20]. To identify whether the observed disorganisation of the outer cell wall layer was a result of increased chitin incorporation, the cell wall of acidic adapted cells was stained with Calcofluor White (CFW). Quantification of CFW fluorescence indicated that the chitin content of the cell wall was only elevated at pH2 (Fig 2A), a result that was confirmed by HPLC (Table 1), suggesting that adaptation to environments of very low pH requires chitin synthesis. However, staining the cell wall with wheat germ agglutinin (WGA), a lectin that binds surface exposed chitin, indicated that even adaptation to pH4 required reorganisation of cell wall chitin, with chitin becoming increasingly exposed during adaptation to acidic pH (Fig 2B). Microscopy confirmed that cells adapted to acidic pH showed significant de-cloaking of chitin around the cell periphery, with intense WGA staining occurring at bud scars at pH2 (Fig 2C). To deduce whether this de-cloaking phenomenon is an active process, we analysed the de-cloaking of chitin in response to pH in dead cells. De-cloaking of cell wall chitin only occurred in live cells (S1 Fig), confirming that the observed increase in surface exposure of chitin was not a physical effect of the pH simply degrading the cell wall. Thus, adaptation to acidic environments is a two-stage process with moderate acidic environments (pH4) causing active de-cloaking of chitin, and strong acidic environments (pH2) inducing *de novo* chitin synthesis and further de-cloaking of chitin.

**Table 1:** Relative dry weight proportions (%) of glucan and chitin in the cell wall quantified by HPLC.

Condition	Chitin	Glucan
pH2	21.11 (± 3.71)	78.89 (± 3.71)
pH4	10.69 (± 9.77)	89.31 (± 9.77)
pH6	7.97 (± 0.83)	87.26 (± 7.58)
pH8	7.73 (± 5.42)	88.02 (± 0.51)

### Neither Mkc1, Hog1, or Crz1 regulate chitin exposure in response to moderate pH stress

**Fig 3:**
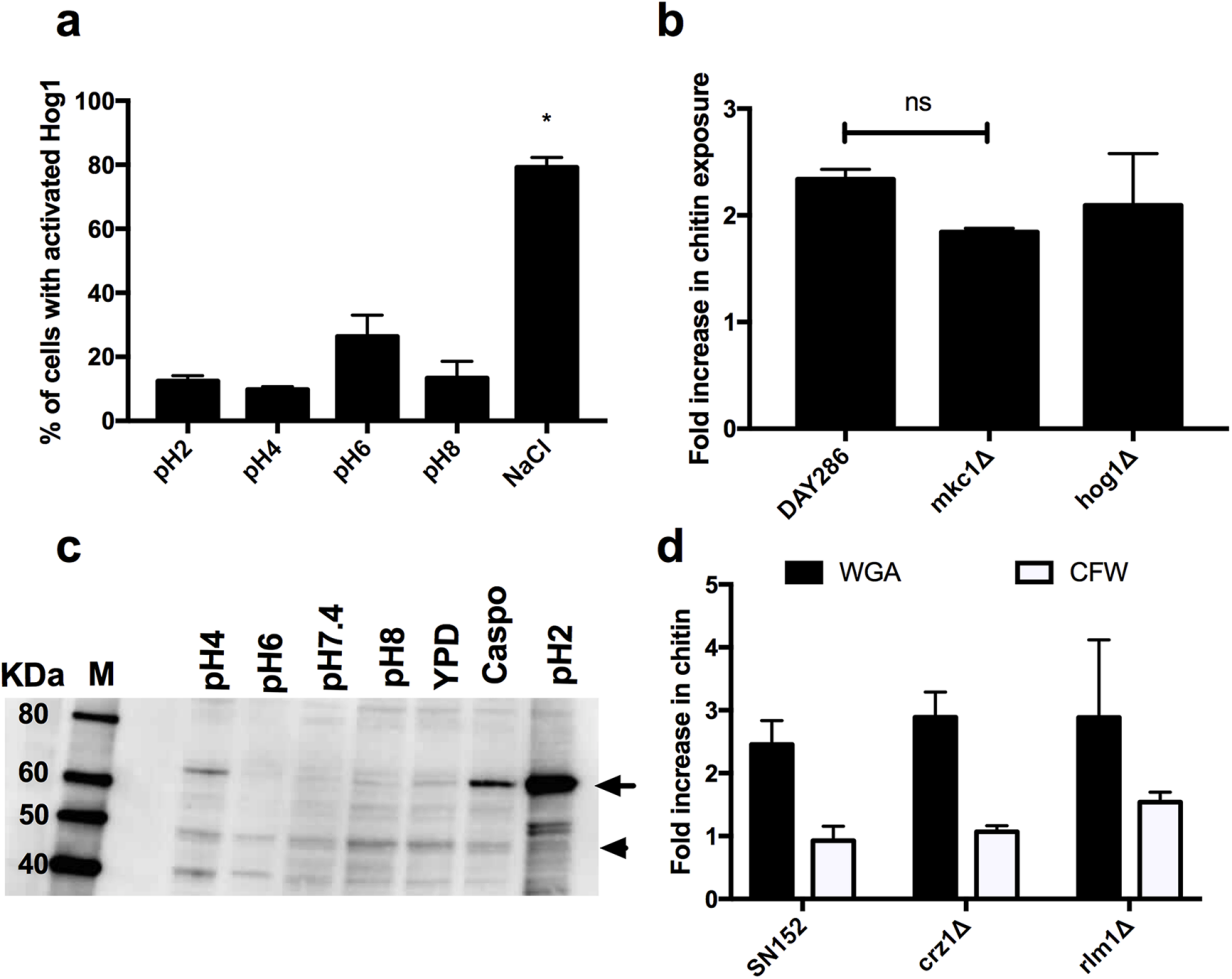
Mkc1, Hog1 and Crz1 are not required for de-cloaking of chitin in response to acidic pH. **a)** A *C*. *albicans* strain expressing Hog1-GFP was grown in YPD buffered at the appropriate pH for 4 h and the localisation (cytoplasmic vs. nuclear) of Hog1 scored in 200 cells per condition. As a positive control of Hog1 activation, cells grown in YPD were incubated with 1 M NaCl for 30 min prior to imaging. The data represent the mean ± SEM from three independent experiments. **b)** Increase in WGA and CFW staining in kinase mutants grown at pH4 relative to YPD as quantified by FACS analysis. Data represent the mean ± SEM from three independent experiments. DAY286 is the parental control strain of the two kinase mutants **c)** Wild type *C*. *albicans* (NGY152) was grown to mid-log phase in YPD buffered at the appropriate pH, total protein extracted and activation of Mkc1 and Cek1 assessed via western blot using the MAPK p42/p44 antibody which cross reacts with both kinases. Arrow indicates Mkc1, while arrowhead indicates Cek1. **d)** Increase in WGA and CFW staining in transcription factor mutants at pH4 relative to YPD as quantified by FACS analysis. SN152 is the parental control strain of the two transcription factor mutants. Data represent the mean ± SEM from three independent experiments.

Chitin synthesis is regulated by the cell wall salvage, calcium/calcineurin and high osmolarity glycerol (HOG) signalling pathways [21]. To assess whether these pathways are involved in chitin reorganisation, activation of these signalling cascades in response to environmental pH was determined. Hog1 was not activated during exponential growth in environments of differing pH (Fig 3A). In agreement with this, deletion of *HOG1* did not impact on the surface exposure of chitin during adaptation to pH4 environments (Fig 3B). Although the cell wall salvage pathway was activated in response to acidic environments (Fig 3C), deletion of *MKC1* and *RLM1* did not prevent the cell wall remodelling that results in de-cloaking of the underlying chitin (Fig 3B and 3D). Deletion of *CRZ1*, the transcription factor downstream of the calcium/calcineurin pathway, also had no impact on chitin exposure in acid adapted *C*. *albicans* cells (Fig 3D). Therefore, these pathways are not involved in the reorganisation of chitin during adaptation to pH4 environments.

### Rim101 and Bcr1 coordinate correct localisation of chitin in the cell wall through regulation of Cht2

**Fig 4:**
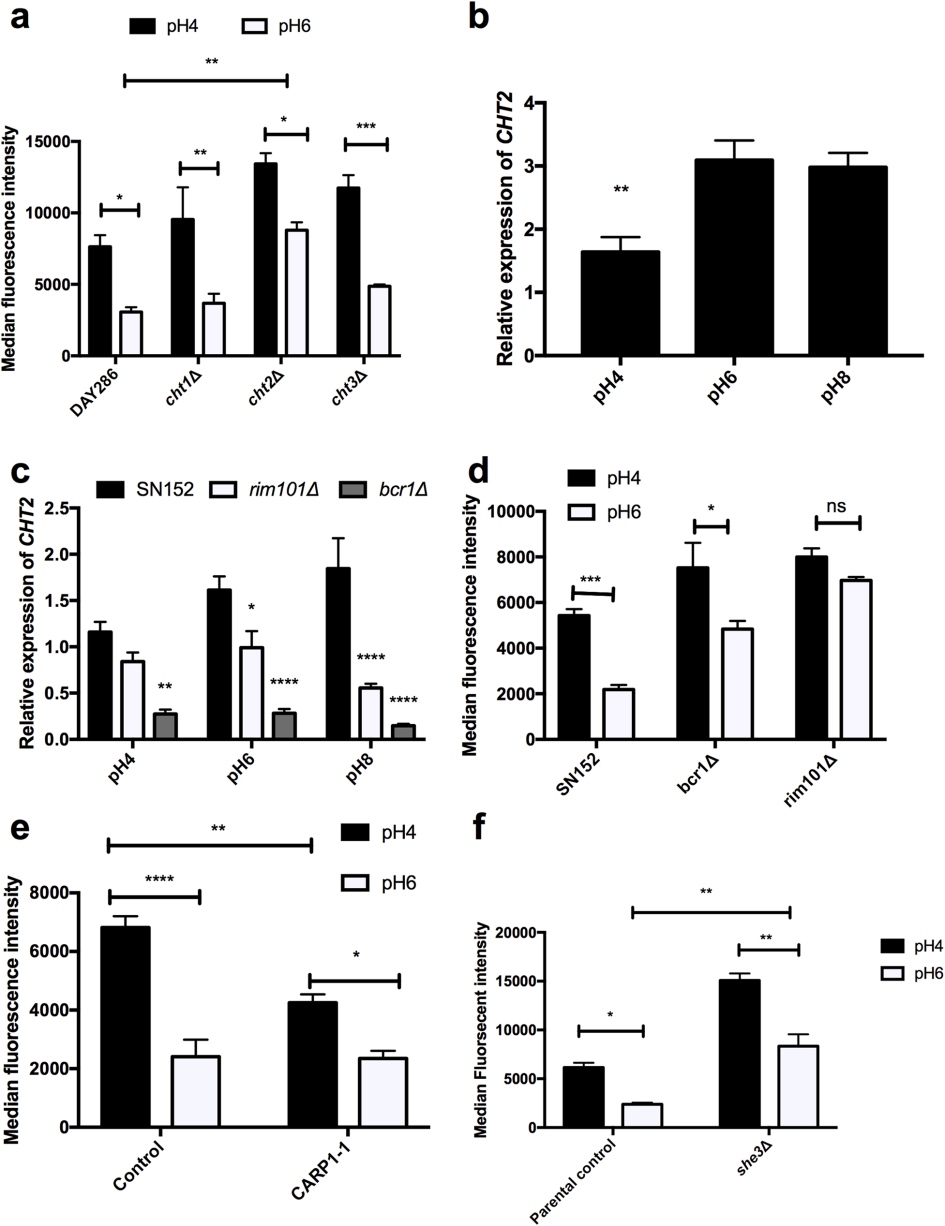
Masking of chitin in the cell wall is regulated by Rim101 and Bcr1 and requires Cht2. **a)** Respective strains were grown in YPD buffered at pH4 or pH6 to mid-log phase, cells were fixed, stained with FITC-WGA and fluorescence quantified by FACS. **b)** SC5314 was grown to mid-log phase in YPD buffered at pH4, pH6 and pH8, snap frozen and total RNA extracted. Expression of *CHT2* was determined by semi quantitative RT-PCR using 50 ng of total RNA. Expression levels were normalized to *ACT1*. **c)** Relative expression of *CHT2* in *rim101*Δ and *bcr1*Δ mutants exponentially growth in YPD buffered at pH4, pH6 and pH8 and determined by semi quantitative RT-PCR. Expression levels were normalized to *ACT1*. **d)** FACS analysis of WGA staining in *rim101*Δ and *bcr1*Δ mutants. **e)** FACS analysis of WGA staining in CARP1-1 which expresses constitutively active Rim101 **f)** FACS analysis of WGA staining in *she3*Δ mutants. Data represent the mean ± SEM from three independent experiments. (**** p < 0.0001, *** p < 0.001, ** p < 0.01, * p < 0.05).

Chitin can be remodelled in the cell wall through the actions of four chitinases (Cht1-4) [22]. Therefore, the role of these chitinase enzymes in the de-cloaking of chitin in response to environmental pH was determined. Deletion of *CHT2* resulted in enhanced chitin exposure at pH6 (Fig 4A) compared to parental control strains. To determine whether the expression of *CHT2* is regulated by ambient pH, semi-quantitative RT-PCR was performed. *CHT2* expression was repressed in acidic environments compared to pH6 or pH8 environments (Fig 4B). Large scale transcriptional profiling suggests that expression of *CHT2* is largely dependent on the transcription factor Bcr1 [23]. To confirm this, the expression of *CHT2* in a *bcr1* mutant strain was determined. *CHT2* expression was significantly reduced in the *bcr1* mutant at all pH conditions tested (Fig 4C), confirming a dominant role for Bcr1 in the expression of *CHT2*.

To investigate the mechanism by which *CHT2* is repressed during adaptation to acidic environments, we focused on the Rim101 transcription factor. Rim101 has previously been shown to regulate the expression of the cell wall modifying enzymes Phr1 and Phr2 in response to environmental pH [24], has been implicated in cell wall reorganisation [25], and is an activator of gene expression in alkaline environments [26]. The expression of *CHT2* was repressed in the *rim101*Δ mutant in environments above pH6 (Fig 4C), with *CHT2* expression levels at pH6 or pH8 being comparable to pH4, suggesting that Rim101 is a pH-dependent activator of *CHT2* expression. Alkaline induced expression of *CHT2* was dependent on Bcr1, as *CHT2* levels remain low under all pH conditions in the *bcr1*Δ mutant (Fig 4C).

To determine how the deregulation of *CHT2* expression affects chitin exposure, the *bcr1*Δ and *rim101*Δ mutants were grown in YPD buffered at pH4 and pH6, and stained with WGA. Deletion of *BCR1* resulted in increased chitin exposure at pH6 (p = 0.0076) and pH4 (p = 0.0491), but maintained some pH dependency, while deletion of *RIM101* resulted in constitutively high chitin exposure (Fig 4D). To investigate whether expression of Rim101 at pH4 would affect the phenotype, we quantified chitin exposure in a strain expressing constitutively active Rim101 (CARP1-1). FACS analysis confirmed that expression of active Rim101 in acidic environments reduced chitin exposure (Fig 4E), but was still insufficient to completely mask the chitin. These results confirm that Rim101 plays an important role in the regulation of cell wall genes pivotal to correct chitin incorporation.

The mRNA of *CHT2* is a target of She3, which transports mRNAs to the cell wall [27]. Therefore, we determined whether this complex is also required for correct incorporation of chitin into the cell wall. Deletion of *SHE3* resulted in enhanced de-cloaking of chitin in environments above pH4 (Fig 4F), similar to the *cht2*Δ and *bcr1*Δ mutants. Taken together, these results suggest that incorporation of chitin into the inner cell wall at pH6 is regulated by Rim101 and Bcr1 and requires She3 dependent delivery of Cht2 to the cell wall, while growth in an acidic environment inactivates this pathway leading to reduced *CHT2* expression and enhanced exposure of chitin at the cell surface.

### Low pH promotes unmasking of the underlying β-glucan

**Fig 5:**
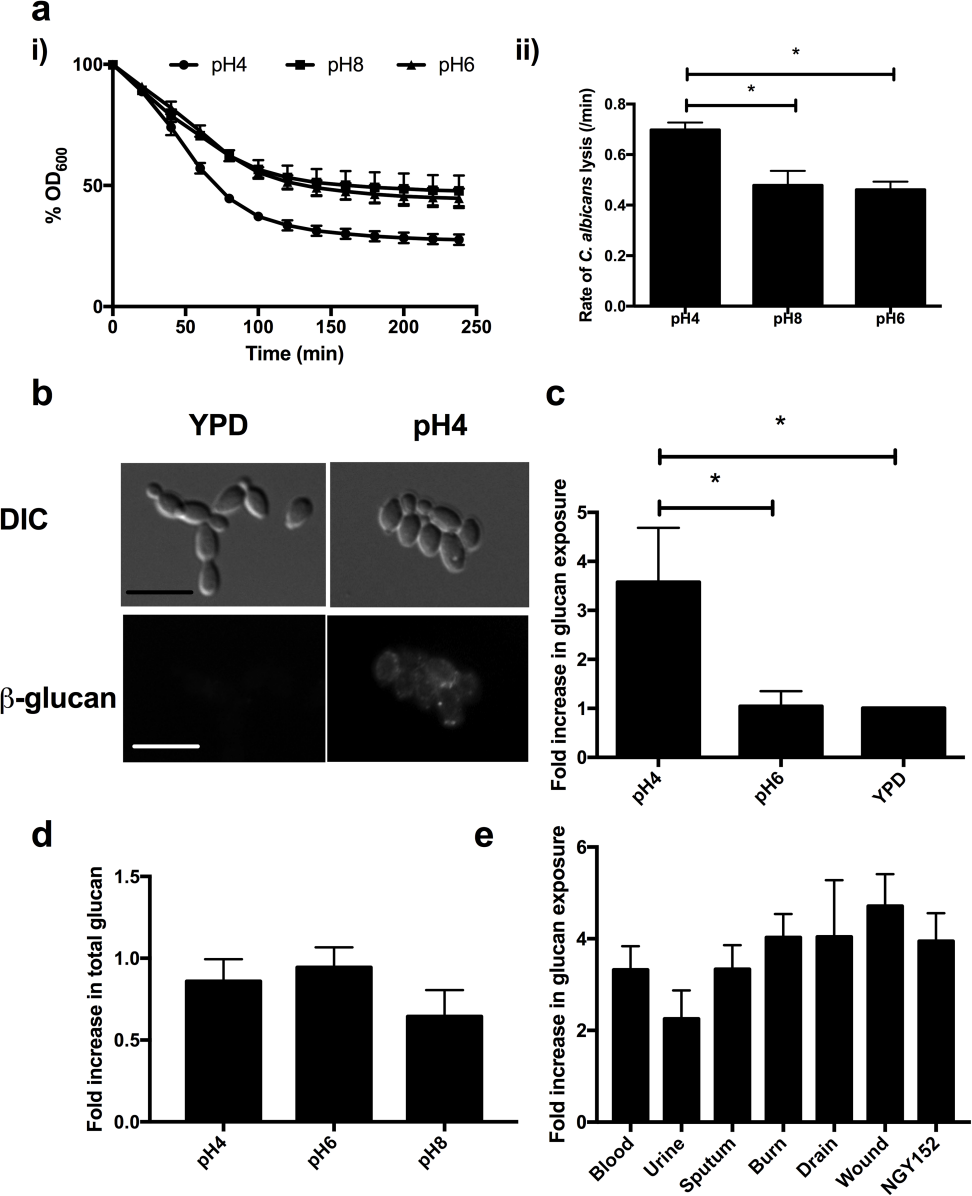
Acidic environments unmask β-glucan in *C*. *albicans*. **a i)** Wild type *C*. *albicans* (NGY152) was grown to mid-log phase in YPD buffered at the appropriate pH, and incubated with recombinant β1,3-glucanase. The decrease in OD_600_ represents cell lysis as the β1,3-glucanase digests the cell wall and is expressed as a percentage of the starting OD_600_. **ii)** The initial rate of cell lysis as calculated from the first 100 min. Data represent the mean ± SEM from four independent repeats **b)** Immunofluorescent imaging of β-glucan exposure of exponentially growing NGY152 cells using a anti-β1,3-glucan monoclonal antibody. Scale bar = 10 μm. **c)** Quantification of β-glucan exposure by FACS counting 10,000 events per repeat. Fold increased is relative to unbuffered YPD. Data represent the mean ± SEM from six independent experiments. **d)** Quantification of Aniline Blue staining of exponentially growing NGY152 cells by FACS analysis counting 10,000 events per repeat. Fold increased is relative to unbuffered YPD. Data represent the mean ± SEM from three independent experiments. **e)** β-glucan exposure of clinical *C*. *albicans* isolates grown to mid-log phase in YPD buffered to pH4 relative to YPD. Data represent the mean ± SEM from three independent experiments (* p < 0.05).

β-glucan is a major component of the cell wall, which is highly immunostimulatory and consequently is normally masked by a dense layer of glycosylated proteins. As a result, fungal cells have a degree of resistance to extracellular glucanases, which break down the cell wall and cause cell lysis. Therefore, the sensitivity of *C*. *albicans* to recombinant β-glucanase can be used as an indirect measure of β-glucan exposure [28]. Growth of *C*. *albicans* in acidic environments enhanced β-glucanase sensitivity (Fig 5Ai and ii) compared to pH6 or pH8 grown cells, suggesting that β-glucan is more accessible in cells grown in media buffered to pH4. In agreement with this, immunofluorescent staining of the cell wall with a monoclonal β1,3-glucan antibody revealed enhanced staining around the cell wall periphery (Fig 5B). Quantification of the immunofluorescent staining by FACS revealed that acid adapted cells had almost 4-fold more surface exposed β-glucan (Fig 5C) than cells grown in YPD media, suggesting that adaptation to acidic environments induces unmasking of β-glucan. To rule out the possibility that the pH simply degrades the cell wall, exposing the underlying β-glucan, dead cells were exposed to YPD at pH2, 4 and 6 for 4 h and surface exposure quantified by FACS. Only live cells unmasked their β-glucan in response to acidic pH (S1 Fig), confirming that, like the chitin exposure, unmasking of β-glucan is an active process.

To determine whether β-glucan synthesis was required for this unmasking effect, total glucan levels were quantified by staining the cells with Aniline Blue. Binding of Aniline Blue to the fungal cell wall was consistent across all pH conditions, suggesting that total β-glucan levels remained constant (Fig 5D). Furthermore, HPLC analysis confirmed that the amount of glucose in the cell wall was not significantly different in cells grown in media buffered to different pHs (Table 1). Therefore, the increase in β-glucan exposure occurs as a result of cell wall remodelling, and not enhanced β-glucan synthesis.

To deduce whether de-cloaking of the cell wall was a general adaptation response of *C*. *albicans*, pH-dependent cell wall reorganisation of a series of clinical isolates from different sites and types of infection was examined. All clinical isolates de-cloaked their cell wall in response to acidic environments (Fig 5E), confirming that this phenomenon is not restricted to laboratory-evolved strains.

**Fig 6:**
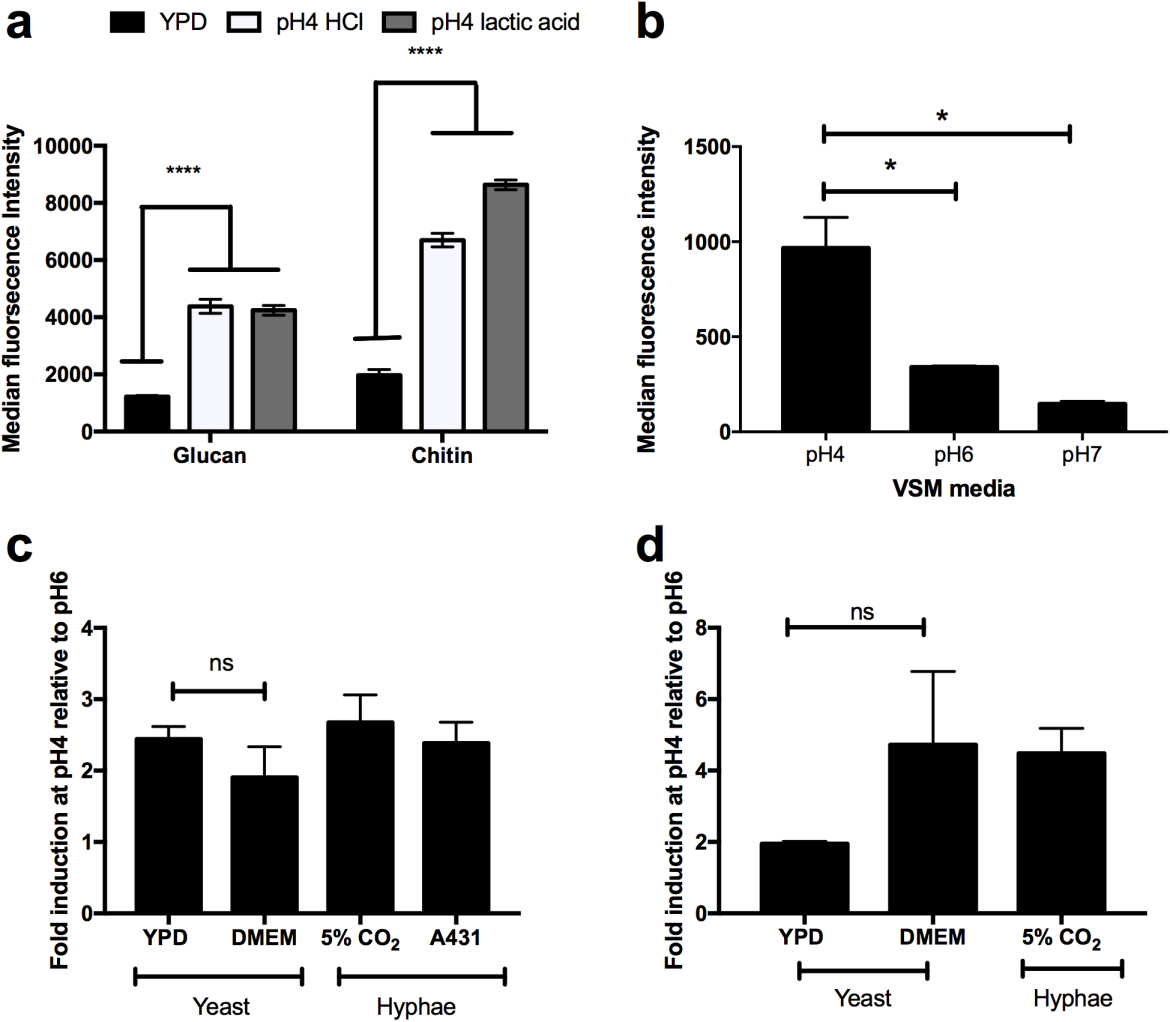
pH-dependent cell wall remodeling occurs in response to physiologically relevant acids and is not restricted to yeast cells. **a)** *C*. *albicans* cells (NGY152) were grown in YPD, or YPD buffered to pH4 by the addition of HCl or lactic acid to mid-log phase, fixed with 4% PFA, stained for β-glucan and chitin exposure and fluorescence intensity quantified by FACS. Data represent the mean ± SEM from three independent repeats (**** p < 0.0001). **b)** *C*. *albicans* cells (NGY152) were grown in vaginal secretion medium (VSM) buffered at pH4, pH6 or pH7 for 4 h, fixed, stained for β-glucan and fluorescence intensity quantified by FACS. Data represent the mean ± SEM from six independent repeats (**** p < 0.01). **c)** *C*. *albicans* cells (NGY152) were grow in 24-well plates in either YPD buffered at pH4 and pH6, DMEM buffered at pH4 and pH6 at 37C, 150 rpm, or grown in DMEM buffered at pH4 or pH6 with 5% CO_2_ in the presence or absence of A431 vaginal epithelial cells for 4 h. Cells were fixed in 4% PFA and stained for β-glucan and **d)** chitin exposure. Results are fold increase relative to pH6. Data represent the mean ± SEM from three independent repeats.

To investigate whether the form of acid used to pH the media affects unmasking, YPD media was buffered using lactic acid an organic acid produced by Lactobacilli during colonisation of the vaginal mucosa. Growth in YPD buffered to pH4 with lactic acid still induced unmasking of the cell wall, similar to reducing pH with HCl (Fig 6A). Therefore, physiologically relevant acids drive unmasking of the cell wall in a pH-dependent manner. In order to determine whether environments that more closely mimic the environment of the female reproductive tract induce cell wall remodelling, *C*. *albicans* was grown in Vaginal Simulation Media (VSM) buffered to pH4, pH6 or pH7. *C*. *albicans* unmasked significantly more β-glucan (p = 0.0022) when the VSM was buffered to pH4 than either pH6 or pH7 (Fig 6B), suggesting that the environment within the female reproductive tract has the potential to induce exposure of β-glucan. Because infection sites are known to contain a mixture of yeast and hyphal cells, *C*. *albicans* hyphae were generated in acidic media in elevated concentrations of carbon dioxide, a potent inducer of morphogenesis [29] and in the presence of vaginal epithelial cells. Both yeast and hyphal cells showed increased chitin and β-glucan exposure compared to their respective controls (Fig 6C and 6D). Therefore, both yeast and hyphal cells undergo cell wall remodelling during adaptation to acidic environments, which results in the surface exposure of β-glucan.

**Fig 7:**
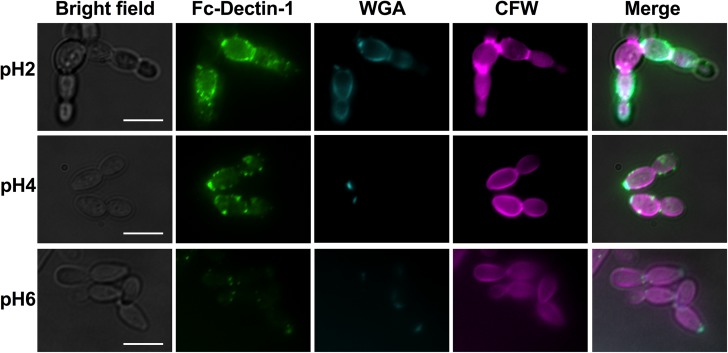
Exposure of β-glucan and chitin do not co-localise. Wild type *Candida albicans* cells (NGY152) were grown to mid-log phase in YPD buffered at pH2, pH4 and pH6, washed, fixed with 4% PFA and stained with CFW, TRITC-labeled WGA, and Fc-Dectin-1 (FITC). White arrowheads indicate patches of β-glucan exposure. Scale bar represents 10 μm.

As adaptation to acidic pH resulted in surface exposure of both chitin and glucan we investigated whether these carbohydrates became exposed at the same or different points in the cell wall. Dual immunofluorescent imaging using TRITC-WGA and FITC-glucan, confirmed that while chitin exposure occurred mainly at bud scars at pH4 with increase exposure occurring in the lateral cell wall at pH2, glucan exposure was continuously localised to punctate patches around the cell periphery (Fig 7). As the exposed carbohydrates were not co-localised we hypothesised that they are not dependent on each other, and may be regulated via different processes.

One potential hypothesis to explain the unmasking response to acidic pH is that the chemical cleavage of the outer phosphomannan could reduce the mannan complexity and result in a more porous outer mannan shield, permitting access to the underlying cell wall layers. To test this hypothesis, carbohydrate exposure after adaptation to acidic pH in the *mnn4*Δ mutant, which is devoid of phosphomannan [30], was examined. The *mnn4*Δ mutant de-cloaked its cell wall similarly to the parental control strain (S2 Fig), suggesting that this phenomenon is not a result of the loss of the phosphomannan and likely involves significant active cell wall reorganisation.

### *Candida tropicalis* also unmasks β-glucan in response to acidic pH

**Fig 8:**
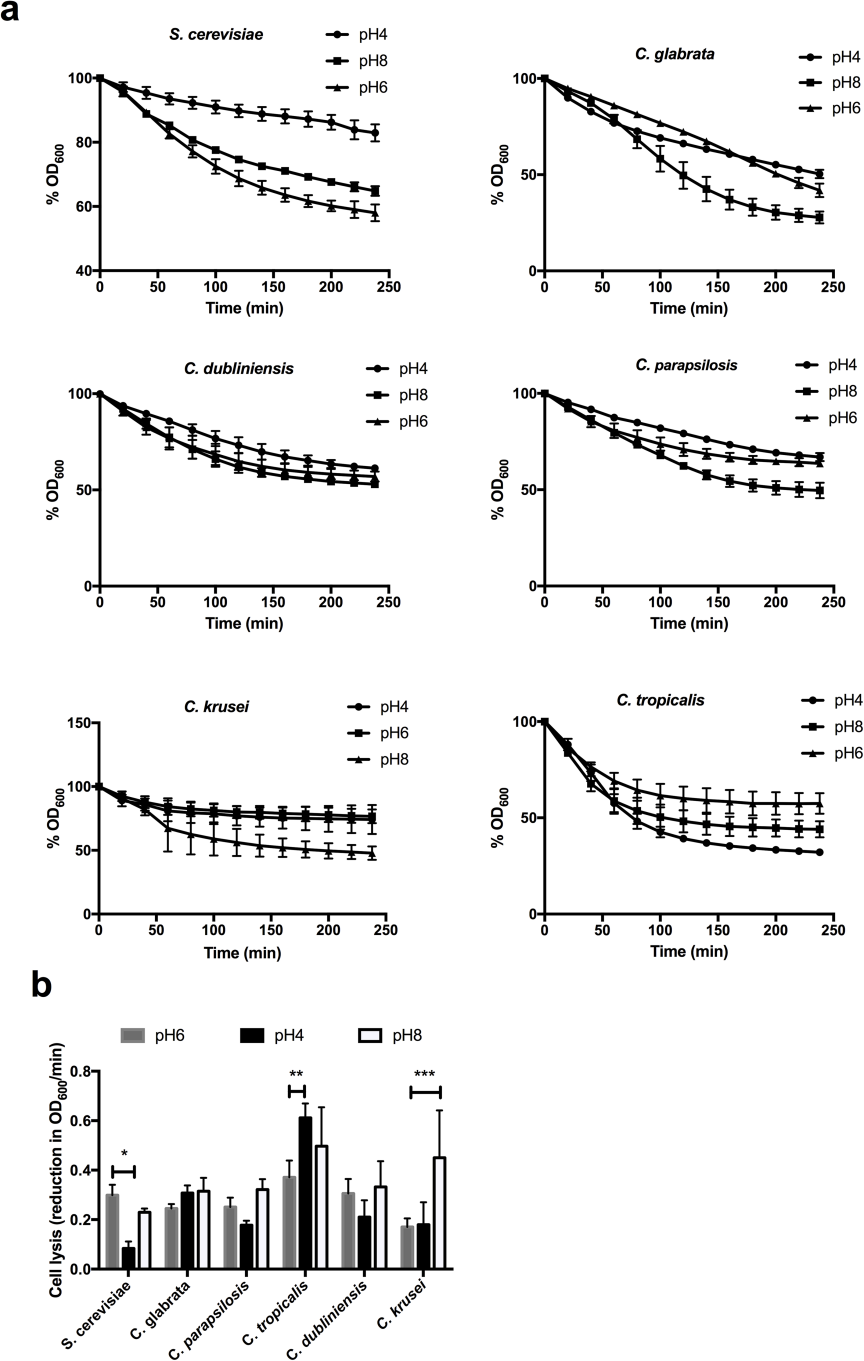
Unmasking of β-glucan in response to environmental pH is specific to *C*. *albicans* and *C tropicalis*. **a)** Exponentially growing cells in YPD, YPD buffered to pH4 and YPD buffered to pH8 were incubated with recombinant β1,3-glucanase. The decrease in OD_600_ represents cell lysis as the β1,3-glucanase digests the cell wall and is expressed as a % of the starting OD_600_. Data represent the mean ± SEM from four independent experiments. **b)** Initial rate of cell lysis was calculated from the first 100 min after the addition of β-glucanase. Data represent the mean ± SEM from four independent repeats (* p < 0.05, ** p < 0.01).

To determine whether unmasking of β-glucan is specific to *C*. *albicans*, the impact of acidic environments on non-*albicans Candida* species and on *Saccharomyces cerevisiae* was assessed. In stark contrast to *C*. *albicans*, growth in acidic conditions reduced the sensitivity of *S*. *cerevisiae* to β1,3-glucanase, suggesting that in response to acidic environments *S*. *cerevisiae* masks its β-glucan (Fig 8), which has been previously reported [28]. Likewise, *Candida parapsilosis* also displayed a mild, statistically insignificant, decreased sensitively to β1,3-glucanase when grown under acidic conditions (Fig 8). On the other hand, *Candida glabrata* and *Candida dubliniensis* did not show any pH-dependent modulation of β-glucan exposure (Fig 8). Unexpectedly, *Candida krusei* unmasked its β-glucan in response to alkaline environments (Fig 8), while *Candida tropicalis* unmasked its β-glucan in response to low pH (Fig 8). Therefore, of the isolates examined only *C*. *albicans* and *C*. *tropicalis* unmask β-glucan in response to low pH.

### Unmasking of β-glucan involves a non-canonical cell wall remodelling pathway

Investigation of the major signal transduction pathways known to be involved in cell wall remodelling (i.e. Mkc1, Hog1, Crz1) confirmed that these pathways are not required for pH-dependent β-glucan unmasking (S3 Fig). Due to the involvement of Rim101 and Bcr1 in chitin de-cloaking, we hypothesised that these transcription factors may also be involved in β-glucan unmasking. However, *rim101*Δ and *bcr1*Δ mutants still unmasked β-glucan in a pH-dependent manner (S3 Fig). As Rim101 does not undergo C-terminal processing at acidic pH, unmasking of β-glucan in a strain constitutively expressing active Rim101 (CARP1-1) was also assessed. The CARP1-1 strain still displayed pH-dependent unmasking of β-glucan (S3 Fig), suggesting that the Rim101 signalling cascade is not required for pH-dependent β-glucan unmasking. Therefore, while Rim101/Bcr1 regulate chitin exposure, a non-canonical signalling pathway regulates pH-dependent β-glucan unmasking.

### Acid adapted *C*. *albicans* cells are readily phagocytosed and induce a strong innate immune response

**Fig 9:**
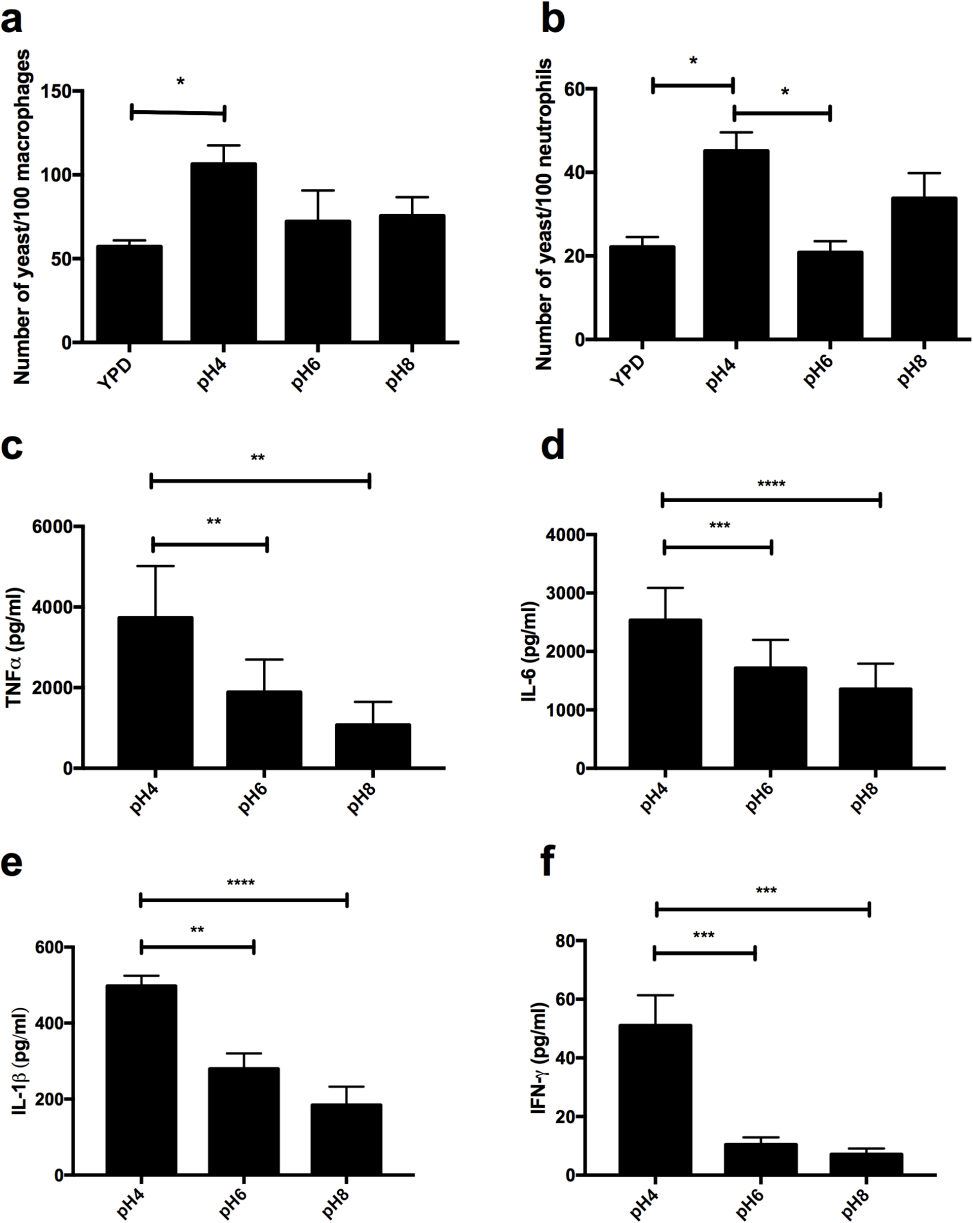
Adaptation to acidic environments increases immune recognition. *C*. *albicans* was grown in YPD at the appropriate pH to mid-log phase, co-incubated with **a)** J774.1A macrophages **b)** neutrophils at an MOI = 5 for 1 h and the rate of phagocytosis determined. Data represent the mean ± SEM from four independent repeats. PBMCs were incubated with PFA fixed mid-log phase wild type *C*. *albicans* cells (NGY152), at an MOI of 0.5 for 24 h. Cytokine secretion was quantified by ELISA, **c)** TNFα, **d)** IL-6, **e)** IL-1β, **f)** IFNγ. Data represent the mean ± SEM from six donors (* p < 0.05, ** p < 0.01, *** p < 0.001, **** p < 0.0001).

The cell wall is the first point of contact between the fungus and the host’s immune system and thus the cell wall plays a major role in innate immune recognition of fungi. Therefore, the role of the pH-dependent cell wall de-cloaking in regulating innate immune responses was investigated. *C*. *albicans* cells grown in acidic media were more readily phagocytosed by macrophages and neutrophils than *C*. *albicans* cells grown in standard YPD media (Fig 9A and 9B).

To deduce whether adaptation of *C*. *albicans* to low pH results in a heightened pro-inflammatory innate immune response, the cytokine response of peripheral blood monocytes (PBMCs) exposed to *C*. *albicans* adapted to different environmental pH conditions was examined. *C*. *albicans* adapted to acidic environments elicited a much stronger proinflammatory cytokine response from PBMCs than *C*. *albicans* cells grown in more alkaline conditions (Fig 9C–9F). Specifically, *C*. *albicans* cells grown in YPD buffered to pH4 induced higher secretion of TNFα, IFN-γ, IL-6 and IL-1β compared to cells grown in YPD buffered to pH6 or pH8. Therefore, de-cloaking of the fungal cell wall in response to acidic pH promotes innate immune recognition of *C*. *albicans*.

The *rim101*Δ and *bcr1*Δ mutants displayed levels of chitin exposure at pH6 similar to those observed at pH4. As chitin has been shown to supress the innate immune system [31, 32], we tested whether the increased chitin in these mutants affected the innate immune response. Phagocytosis rates in both mutants were reduced compared to the parental control strain (S4 Fig). However, this did not impact on the cytokine response (S4 Fig). Therefore, the altered cell wall structure in these mutants affects phagocytosis, but is not sufficient to affect pro-inflammatory cytokine responses.

### Enhanced phagocytosis is mediated by recognition of β-glucan by Dectin-1

**Fig 10:**
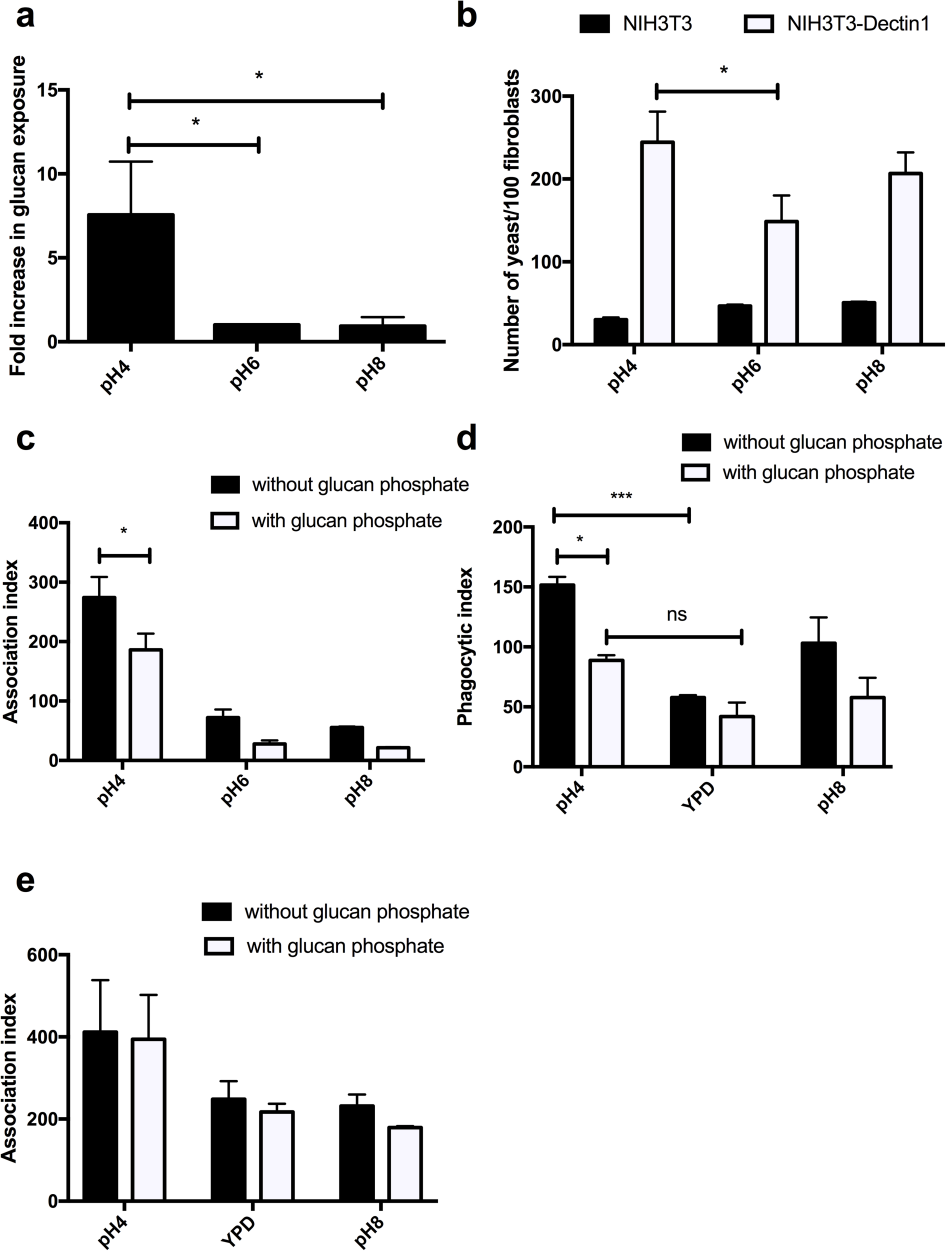
Enhanced immune recognition of acidic adapted cells is mediated via Dectin-1. **a)** Fc-Dectin-1 binding to wild type *C*. *albicans* cells grown to mid-log phase in YPD buffered to the appropriate pH, as quantified by FACS counting 10,000 events per repeat. Fold increased is relative to unbuffered YPD. **b)** *C*. *albicans* was grown in YPD at the appropriate pH to mid-log phase, co-incubated with fibroblasts for 1 h, fixed and the association index (number of fungal cells either attached and phagocytosed/100 macrophages) determined. **c)** Association of acidic adapted *C*. *albicans* cells to fibroblasts expressing Dectin-1 in the presence of glucan phosphate. **d)** J774.1A macrophages were pre-incubated with glucan phosphate and infected with *C*. *albicans* at an MOI of 5 and the phagocytosis index (number of fungal cells phagocytosed/100 macrophages) determined after 1 h. **e)** J774.1A macrophages were pre-incubated with glucan phosphate and infected with *C*. *albicans* at an MOI of 5 and the association index determined after 1 h. Data represent the mean ± SEM from three independent repeats (* p > 0.05. *** p > 0.001).

β-glucan is recognised by the C-type lectin-like receptor Dectin-1 [33]. As the monoclonal anti-β1,3-glucan specific antibody confirmed that growth in acidic conditions resulted in β-glucan unmasking, the accessibility of this exposed β-glucan to Dectin-1 was assessed. Immunofluorescent staining of acid grown cells using Fc-Dectin-1 confirmed that Dectin-1 more readily bound to cells grown in acidic conditions (Fig 10A). To ascertain whether this enhanced binding of Dectin-1 to the surface of acid grown cells resulted in the increased phagocytosis rate, Dectin-1 was expressed on the surface of fibroblasts. Fibroblasts expressing Dectin-1 more readily bound *C*. *albicans* cells grown in acidic conditions than YPD or pH8 grown cells (Fig 10B). Enhanced adhesion was not observed on fibroblasts which did not express Dectin-1, and could be blocked using glucan phosphate (Fig 10C), confirming that the enhanced adhesion is due to a specific interaction between Dectin-1 and surface exposed β-glucan, and not due to differences in electrochemical properties of the cell wall.

To further confirm the role of Dectin-1, the Dectin-1 receptor was blocked with glucan phosphate. Blocking of Dectin-1 in the J774.1A macrophage cell line, resulted in a pH-dependent decrease in phagocytosis (Fig 10D), but did not affect the association of *C*. *albicans* with the macrophage, with acid adapted cells still displaying enhanced association (Fig 10E). Therefore, Dectin-1 is required for the enhancement of phagocytosis, but other pattern recognition receptors are responsible for the initial attachment of acid-adapted *C*. *albicans* to the surface of innate immune cells.

### Adaptation to acidic pH results in increased immune cell recruitment *in vivo*

**Fig 11:**
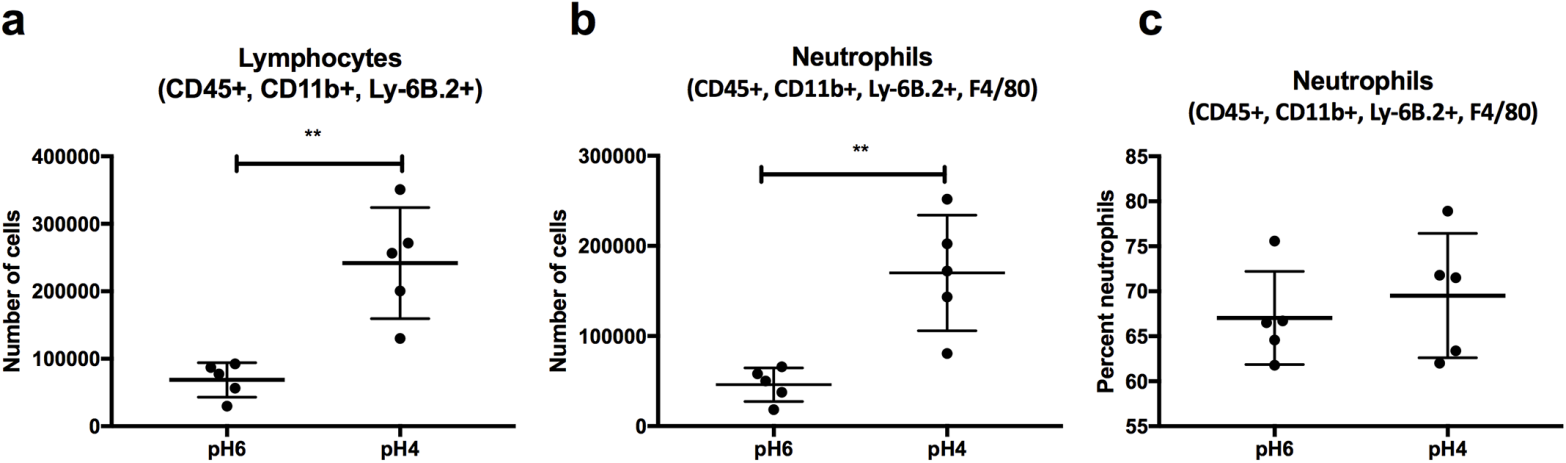
*C*. *albicans* cells adapted to acidic environments recruit more innate immune cells *in vivo*. **a)** Total number of CD45+, CD11b+ and Ly-6B.2+ (7/4 clone) cells (including monocytes and neutrophils) recruited to the peritoneal cavity following 4 h exposure to *C*. *albicans* incubated in pH6 or pH4 YPD (p = 0.007). **b)** Total number of neutrophils (further identified using F4/80) recruited to the peritoneal cavity (p = 0.010) **c)** Percentage of neutrophils in the total population of recruited innate immune cells (p = 0.540).

Assessment of the innate immune response to acid adapted *C*. *albicans* cells confirmed that the increased exposure of glucan results in a heightened proinflammatory immune response. To investigate the *in vivo* significance of this discovery, live *C*. *albicans* cells adapted to different pH conditions were injected into the peritoneal cavity of mice, and the recruitment of innate immune cells was determined after 4 hours. *C*. *albicans* cells adapted to acidic environments recruited more CD45+ lymphocytes than *C*. *albicans* cells adapted to pH6 (Fig 11A, p = 0.007), including significantly more neutrophils than *C*. *albicans* cells adapted to pH6 media (Fig 11B, p = 0.010). However, analysis of the overall cell population confirmed that although the total number of recruited immune cells was increased, there was no significant difference between the percentages of each cell type recruited (Fig 11C, p = 0.540). Therefore, *C*. *albicans* cells adapted to acidic environments initiate a stronger proinflammatory innate immune response, and recruit significantly more immune cells to the site of infection.

## Discussion

*C*. *albicans* has a remarkable ability to respond and adapt to a multitude of environmental signals. Here, we demonstrate that adaptation to low pH results in significant remodelling of the *C*. *albicans* cell wall. The most striking effects include the de-cloaking of the underlying chitin and β-glucan. Unmasking of these underlying carbohydrate epitopes was an active process, and was independent of phosphomannan loss, suggesting that chemical cleavage of this outer cell wall component did not sufficiently deplete the outer mannan armour to reveal internal pathogen associated molecular patterns (PAMPs).

**Fig 12:**
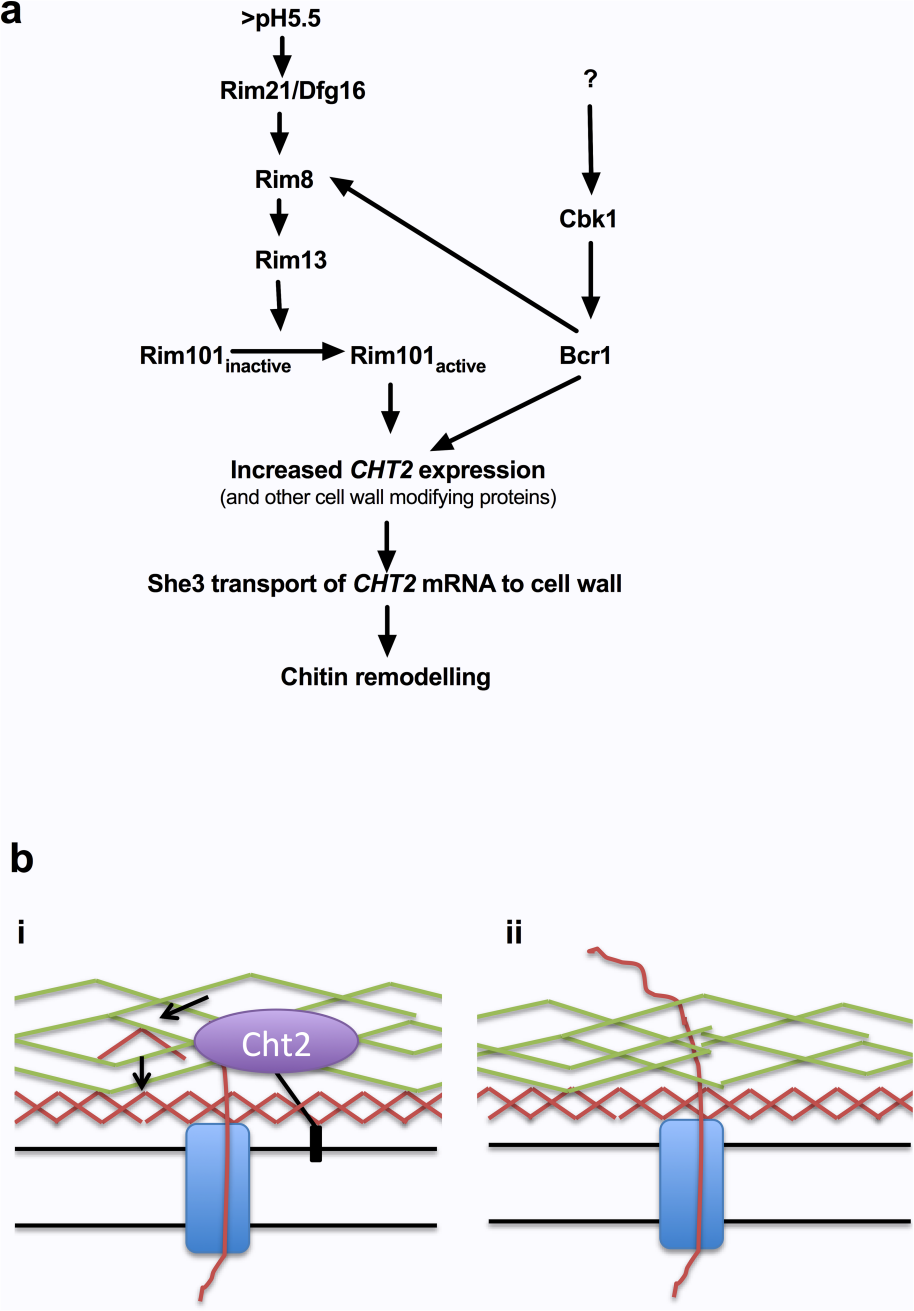
Regulation of cell wall remodeling during adaptation to environmental pH. **a)** In environments above pH5.5, the Rim101 pathway is activated resulting in C-terminal processing of Rim101 and enhance expression of *CHT2*. The She3 complex then transports *CHT2* mRNA to the cell wall, where it is translated and attached via its GPI anchor. At pH4 Rim101 is not activated, resulting in reduced expression of Cht2. **b) (i)** When present in the cell wall (i.e. above pH5.5), Cht2 hydrolyses the growing chitin polymer into shorter fragments which may hydrogen bond and form short chitin microfibrils that are embedded deep within the cell wall. **(ii)** When there is reduced amounts of Cht2 in the cell wall (i.e. environments below pH4), the growing chitin polymer is cleaved less efficiently, resulting in the incorporation of longer chitin polymers that are more exposed on the outer surface of the cell wall.

Chitin is remodelled in the cell wall through the actions of chitinases. *C*. *albicans* expresses four chitinases [22], however only deletion of *CHT2* prevented the pH-dependent de-cloaking of chitin. Cht2 is mainly expressed in yeast cells, and is GPI-anchored to the cell wall [34]. pH-dependent de-cloaking of cell wall chitin also was dependent on the Rim101 and Bcr1 transcription factors. Rim101 is C-terminally processed in response to alkaline environments through the *RIM101* signalling cascade, and activates the expression of alkaline induced genes, while repressing the transcription of acid induced genes [26]. Bcr1 is essential for biofilm formation and controls the expression of many cell wall associated genes including *HWP1*, *ALS1*, *ALS3* and *HYR1* [35]. The expression of *CHT2* was dependent on Bcr1, and showed Rim101 pH-dependent expression in environments above pH6. This alkaline dependent increase in expression was lost in the *bcr1*Δ mutant, suggesting that Bcr1 is essential for *CHT2* expression, as indicated in large scale transcriptomic analyses [36]. However, Bcr1 also positively regulates *RIM8* [37], suggesting that inactivation of *BCR1* would also decrease processing of Rim101, linking the two pathways (Fig 12A).

One hypothesis for the de-cloaking of chitin is that during growth in neutral or alkaline environments Rim101 and Bcr1 activate the expression of *CHT2*. She3 then delivers *CHT2* mRNA to the cell wall, where it is translated, and incorporated into the cell wall via its GPI anchor. Cht2 is predicted to possess endo-chitinase activity. Therefore, once in the cell wall, Cht2 would act internally on the chitin microfibrils, decreasing their length, and permitting correct incorporation in the inner layer of the cell wall. However, during growth in acidic conditions, Rim101 is inactive, resulting in lower transcriptional levels of *CHT2* and other cell wall regulatory enzymes. In this case, there will be less Cht2 to act on the chitin microfibrils, which may increase the length of these microfibrils, hindering their embedment into the inner layer of the cell wall and resulting in increased exposure of chitin at the cell surface (Fig 12B).

More striking than the de-cloaking of chitin was the unmasking of the highly pro-inflammatory PAMP, β-glucan. Dissection of key pathways known to be involved in the regulation of cell wall biosynthesis and pH sensing suggested that these conventional pathways are not responsible for regulating the change in distribution of β-glucan. In *S*. *cerevisiae*, adaptation to acidic environments has opposing effects inducing masking of β-glucan, which is mediated by the Hog1 signal transduction pathway [28]. Deletion of *HOG1* had no impact on β-glucan unmasking in *C*. *albicans*, suggesting that significant transcriptional rewiring has occurred during evolution between *S*. *cerevisiae* and *C*. *albicans*.

*C*. *albicans* is isolated from 95% of vulvovaginal candidiasis (VVC) cases, with the remaining 5% of infections being mainly caused by *C*. *glabrata* [38]. During colonisation of the vaginal mucosa *C*. *albicans* is exposed to many environmental conditions including low mucosal pH (vaginal pH = 3.5–4.5). Unlike bacterial vaginosis, which is associated with alkalisation of the vaginal mucosa, during VVC mucosal pH remains low [39]. Therefore, during both colonisation and infection of the vaginal mucosa, *C*. *albicans* is exposed to acidic conditions.

The pH-dependent unmasking of the cell wall results in enhanced recruitment of innate immune cells *in vivo*, which correlated with enhanced production of proinflammatory cytokines. Our *in vitro* assays confirm that this enhanced immune recognition was mediated via the C-type lectin like receptor Dectin-1, a dominant receptor in fungal innate immunity known to recognise β-glucan [40].

Symptomatic vaginal colonisation by *C*. *albicans* results in excessive neutrophil migration, with vaginal secretions displaying enhanced chemotactic potential and increased mucosal damage [41]. Neutrophil depletion reduces inflammation and symptoms associated with VVC [42], suggesting that neutrophil recruitment in VVC is non-protective, in contrast to oral candidiasis where neutrophil recruitment and activation of Th17 responses are protective [43].

Growth of *C*. *albicans* in VSM (a media which more closely resembled vaginal fluid) confirmed that the unmasking of β-glucan is not media specific, but is pH dependent, with unmasking only occurring at acidic pH. This raises questions regarding the link between β-glucan unmasking and VVC, as mouse VVC models, which elicit similar immunopathologies to human samples, have a neutral, not an acidic, vaginal pH [44]. Although the mouse models can replicate some conditions experienced during VVC, these still do not provide a complete model of VVC. For example, mice are not naturally colonised by *C*. *albicans* in the vaginal tract, and are normally immune suppressed with oestrogen to maintain colonisation and the high inoculum used to establish the infection is unlikely to reflect fungal burdens during the initiation of VVC [44]. The cells used for intravaginal inoculation will also be immunogenically different to those acquired from the GI tract or skin, which is the primary source of vaginal seeding in human VVC. Finally, the neutral pH of the mouse vaginal mucosa would favour rapid hyphal development of *C*. *albicans*, which will activate the inflammasome pathway more rapidly resulting in increased epithelial activation and secretion of proinflammatory cytokines [45]. Therefore, although the mouse model parallels the symptoms of human VVC we still do know whether the observed immunopathology arises from the same underlying mechanism. For example, it has recently been reported that neutrophils also promote unmasking of β-glucan [46], which would also increase inflammation. Therefore, although we have animal models for VVC, these do not currently directly reflect human infection. It is conceivable that in human VVC, a gradual increase in fungal burden in combination with pH-dependent unmasking of the immunostimulatory β-glucan in both yeast and hyphal cells, as a response to environmental changes within the vaginal niche, might initiate a earlier, stronger proinflammatory cytokine response, resulting in increased neutrophil recruitment causing symptomatic vaginal colonisation.

## Materials and methods

### Ethics

All animal work was carried out by competent researchers under UK Home Office project licence PPL 70/9027 (awarded to Dr Donna MacCallum), which was reviewed and approved by the University of Aberdeen Animal Welfare and Ethical Review Body (AWERB) and the UK Home Office. Animal experiments adhered to the UK Animals (Scientific Procedures) Act 1986 (ASPA) and European Directive 2010/63/EU on the protection of animals used for scientific purposes. All animal experiments were designed with the 3Rs in mind and were reported using the ARRIVE guidelines. The Institutional Review Board of the School of Biosciences at the University of Birmingham approved the protocol for blood collection, and isolation of PBMCs and neutrophils from healthy volunteers. Blood donations were anonymous, and all volunteers provided written informed consent for samples to be included in this study.

### Strains, media and growth conditions

Unless stated otherwise, all media and consumables were purchased from Sigma-Aldrich UK. Yeast strains were maintained on YPD agar (1% yeast extract, 1% bacto-peptone, 2% glucose and 2% agar). For liquid cultures, yeasts were cultured in YPD (1% yeast extract, 1% bacto-peptone, 2% glucose). Buffered YPD was made by supplementing YPD with 3.57% HEPES and adjusting the pH accordingly. VSM contained 58 mM NaCl, 18 mM KOH, 2 mM Ca(OH)_2_, 1.75 mM glycerol, 6.7 mM urea, 33 mM glucose, and 0.67% yeast nitrogen base (YNB) and buffered to pH4, pH6 or pH7 with 22 mM lactic acid and 17 mM acetic acid according to [47]. Strains used in the study are listed in S1 Table.

### High pressure freeze substitution transmission electron microscopy

The ultrastructure of the cell wall of wild type (NGY152) cells grown at different pH was visualised by TEM. Exponentially growing cells at 37°C were frozen under high pressure in liquid nitrogen and embedded in resin as described previously [48]. Ultrathin sections (70 nm) were cut and mounted on 400 mesh copper grids and stained in 4.5% uranyl acetate in 1% acetic acid for 45 min and Reynolds lead citrate for 7 min. Images were acquired using a Jeol 1230 at 80 kV accelerating voltage fitted with a Gatan 791 multiscan camera. Five cells were selected at random for each condition and multiple images taken around the cell periphery. The thickness of the inner and outer cell wall layers was measured from at least 30 different points of each cell using ImageJ. Data were analysed by two-way ANOVA followed by post-hoc Tukey’s multiple comparisons test at 95% confidence.

### Immunofluorescent staining of cell wall components

*C*. *albicans* was grown overnight in appropriately buffered YPD at 37°C, 200 rpm. Cells were sub-cultured in fresh media and grown to exponential phase at 37°C, 200 rpm. Cells were harvested by centrifugation at 3500 rpm for 3 min, fixed on ice for 30 min in 4% PFA in PBS and washed three times in PBS. To kill *C*. *albicans* to test whether cell viability is required for cell wall unmasking, overnight cultures of SC5314 were either fixed with 4% PFA for 45 mins, heat killed at 65°C for 2 h, treated with 1 J UV in a UVILink CL-508G cross-linker (UVITec), or treated with 100 mM thimerosal for 45 min. Following killing, *C*. *albicans* cells were washed in PBS and incubated in YPD buffered at pH2, pH4 and pH6 for 4 h. To generate hyphal cells in acidic environments, 2 x10^6^ yeast cells were inoculated into DMEM buffered at pH4 of pH6 and incubated under static conditions in 5% CO_2_ either with or without 2 x10^5^ A431 vaginal epithelial cells (Sigma) in 24 well plates for 4 h. For controls (yeast cells), *C*. *albicans* were incubated in YPD buffered at pH4 or pH6, and DMEM buffered at pH4 and pH6 at 37°C, 150 rpm, in a 24 well plate. Quantification of WGA staining in the presence of vaginal epithelial cells could not be performed due to non-specific binding of the lectin to the epithelial cells.

To stain for surface exposed chitin, 2 x10^6^ cells were incubated with 100 μg/ml FITC conjugated WGA (Molecular Probes, Life Technologies) for 30 min. To stain for total chitin, fixed cells were stained with 3.5 μg/ml CFW for 5 min. To stain for total glucan, PFA fixed cells were incubated with Aniline Blue fluorochrome (Bioscience supplies) for 15 min. To stain for surface exposed β1,3-glucan, cells were blocked with 2% BSA in PBS, for 30 min and then incubated with a monoclonal anti-β1,3-glucan antibody (Bioscience Supplies, Australia) diluted 1:800 in PBS, 2% BSA on ice for 30 min. Cells were washed three times with PBS, and incubated with 1:200 diluted anti-mouse IgG, conjugated to FITC (Invitrogen) on ice for 30 min. Alternatively, PFA fixed *C*. *albicans* cells were stained with 3 μg/ml Fc-Dectin-1 (a kind gift from Prof G. Brown, University of Aberdeen) [49], and goat anti-human IgG Fc, conjugated to Alexa Flour 488 (Invitrogen). To stain for total mannan, 2 x10^6^ cells were incubated with 100 μg/ml ConA conjugated to TRITC (Molecular Probes, Life Technologies) for 30 min and washed with PBS. Cells were imaged using a Nikon Eclipse TE 2000U, Plan Apo 60x/1.40 NA oil DIC objective magnification using the appropriate filter set, or analysed on an Attune FACS machine (50 mW Blue/Violet standard configuration), with 10,000 events observed. CFW and Aniline Blue fluorescence intensities were quantified using the 405 nm laser on the Attune in combination with 603/48 and 650DPL filters, FITC labelled cells were quantified using the 488 nm laser in combination with 530/30 and 555DLP filters, and TRITC fluorescence was quantified using the 488 nm laser in combination with 574/26 and 650DLP filters. The MFI was corrected for background fluorescence and expressed as a ratio compared to YPD grown cells. FACS data were analysed by Kruskal-Wallis test followed with a post-hoc Dunn’s multiple comparisons test at 95% confidence.

### β1,3-glucanase sensitivity assay

Yeast cells were inoculated in YPD and grown overnight at 37°C, 200 rpm. Cells were sub-cultured into YPD buffered at the appropriate pH to an OD_600_ of 0.1 and grown until mid exponential phase at 37°C, 200 rpm. Cells were harvested, washed once in sterile water and resuspended in fresh assay buffer (40 mM 2-mercaptoethanol, 50 mM Tris-HCl pH 7) at an OD_600_ of 1.0 and 190 μl added to triplicate wells of a 96-welled plate. β1,3-glucanase (Sigma-Aldrich, UK) was resuspended in sterile ultra pure water to (2 U/ml) and 10 μl (0.02 units) added to each well. The OD_600_ was recorded every 2 min as a measure of cell lysis, and data expressed as a percentage of the OD_600_ at the initial time point. The rate of cell lysis was determined from the first 100 min and data were analysed by Kruskal-Wallis test followed by a post-hoc Dunn’s multiple comparisons test at 95% confidence.

### HPLC analysis of the carbohydrate component of the cell wall

NGY152 was grown to exponential phase in YPD media, or YPD media buffered at the appropriate pH at 37°C and the carbohydrates from the cell wall extracted as described previously [48]. Lyophilised cell wall material (3 mg) was acid hydrolysed with trifluroacetic acid for 3 h at 100°C, washed, resuspended to 10 mg/ml and diluted 1:2 for HPLC analysis. Data were analysed by two-way ANOVA followed by post-hoc Tukey’s multiple comparison test at 95% confidence.

### Activation of cell wall remodelling pathways

To deduce whether Hog1 was activated during prolonged exposure to different environmental pH, a *C*. *albicans* strain expressing a GFP tagged version of Hog1 was utilised (hAHGI). GFP-Hog1 was grown to exponential phase in YPD at the appropriate pH, fixed and immediately imaged for GFP-Hog1 localisation. The percentage of cells positive for nuclear localisation (and hence activation), of GFP-Hog1 was quantified in ImageJ from 200 cells per condition per repeat. As a positive control for Hog1 activation, cells growing exponentially in YPD were exposed to 1 M NaCl for 30 min prior to fixation and imaging.

To deduce whether the cell wall salvage pathway was activated upon adaption to environmental pH, NGY152 was grown in appropriately buffered YPD media overnight, diluted 1:100 into fresh media at the appropriate pH and grown at 37°C, 200 rpm until exponential phase. As a positive control for Mkc1 activation, 0.032 μg/ml caspofungin was added to exponentially growing YPD cells for 30 min. Cells were harvested by centrifugation, and immediately snap frozen in liquid nitrogen. Pellets were defrosted in 500 μl RE buffer (50 mM HEPES pH 7.5, 150 mM NaCl, 5 mM EDTA, 1% Triton X-100) supplemented with Roche complete proteinase inhibitor cocktail, washed and resuspended in 500 μl RE buffer. Cells were lysed using a bead beater (6 x 2 cycles 6000 rpm) with 5 min between each cycle. Cell lysis was confirmed by microscopy, and lysates cleared by centrifugation. Total protein concentration was determine by Bradford assay in comparison to a BSA standard curve and 15 μg of total protein was separated by SDS-PAGE on a 4–12% NuPAGE Bis-Tris gel. Proteins were transferred on PVDF membrane, which was blocked with 5% BSA in PBST. Activated (phosphorylated) Mkc1 and Cek1 was detected using an anti-phospho-p44/p42 rabbit monoclonal antibody (Cell Signalling technologies) diluted 1:2000 in 5% BSA, PBST. Protein-antibody complexes were detected using an anti-rabbit IgG-HRP antibody (Invitrogen), diluted 1:5000 in 5% BSA, PBST. Membranes were washed in PBST and signal detected using enhanced chemiluminescence (ECL) kit (Bio-Rad) as per the manufacturer’s recommendations.

### qRT-PCR

*C*. *albicans* cells were grown to mid log phase in YPD buffered to either pH4, pH6 or pH8, centrifuged and snapped frozen in liquid nitrogen. Total RNA was isolated using the RNeasy Plus mini kit (Qiagen) according to the manufacturer’s recommendations and 50 ng of total RNA used for qRT-PCR (Brilliant III Ultra-Fast SYBR Green QRT-PCR master mix, Aligent Technologies) using the following primers ACT1-F: CCTACGTGTACTTGTGCAAGGCAA, ACT1-R: AATGTGTTGCCACTCCAGTT, CHT2-F: AATGTGTTGCCACTCCAGTT, CHT2-R CGGTGCATACAACAGTTTGA. *CHT2* expression was normalised to *ACT1* according to the Delta C(t) method. Values represent the mean +/- SEM from three independent experiments.

### Phagocytosis assay

J774.1A macrophages (Sigma-Aldrich, UK) were maintained in DMEM media supplemented with 10% FBS, 100 mM L-glutamine and 100 mM penicillin/streptomycin at 37°C, 5% CO_2_. 1 x10^5^ J774.1A macrophages were seeded onto 13 mm diameter glass coverslips in 24-well plates and allowed to attach for 24 h. Immediately prior to phagocytosis assays, J774.1A macrophages were serum starved in serum free DMEM media for 1 h with 1.5 μg/ml PMA. Yeast cells were inoculated in YPD and grown overnight at 37°C, 200 rpm. Cells were sub-cultured into YPD buffered at the appropriate pH to an OD_600_ of 0.1 and grown until mid-exponential phase at 37°C, 200 rpm. Cells were harvested, washed three times in sterile, endotoxin free PBS (Sigma-Aldrich, UK) and resuspended in PBS to 1 x10^7^ cells/ml. PMA containing media was aspirated from the macrophages and replaced with fresh serum free DMEM media, to which 5 x10^5^ *Candida* cells were added (MOI = 5). Cells were co-incubated for 1 h, non-phagocytosed *Candida* cells were removed by repeated washing with sterile PBS and cells fixed with 4% PFA for 15 min. To block Dectin-1 recognition, cells were incubated with 50 μg/ml glucan phosphate in serum-free media for 1 h prior to addition of *C*. *albicans*. Media was replaced with fresh serum free media containing 5 x10^5^ *C*. *albicans* cells and 50 μg/ml glucan phosphate (Kind gift from Prof. D. Williams, East Tennessee State University), and cells co-incubated for 1 h. To distinguish between attached and phagocytosed yeasts, coverslips were stained for 30 min with 50 μg/ml ConA conjugated to TRITC (Molecular Probes, Life Technologies), washed three times with PBS and imaged using a Nikon TE2000. At least six images were taken per sample with approximately 100 macrophages/image. Phagocytosis was scored in ImageJ. *Candida* cells stained with ConA-TRITC were considered attached to the exterior of the macrophage while, non-stained yeasts were considered as internalised and phagocytosed. Data were analysed by Kruskal-Wallis test followed with a post-hoc Dunn’s multiple comparisons test at 95% confidence.

### Fibroblast association assay

To assess attachment of *C*. *albicans* to fibroblasts (NIH3T3) or fibroblasts expressing human Dectin-1 (NIH3T3-Dectin-1, kind gift from Prof G. Brown, University of Aberdeen) [50], 1 x10^5^ fibroblasts were seeded on glass coverslips and allowed to attach for 24 h at in DMEM supplemented with 10% FBS, 100 mM L-glutamine and 100 mM penicillin/streptomycin 37°C, 5% CO_2_. Immediately prior to the association assay, fibroblasts were serum starved in serum free DMEM media for 1 h. *C*. *albicans* adapted to different pH conditions were added to the fibroblasts at an MOI = 5. Cells were co-incubated for 1 h, non-attached *Candida* cells were removed by repeated washing with sterile PBS and cells fixed with 4% PFA for 15 min. To block Dectin-1 recognition, cells were incubated with 50 μg/ml glucan phosphate in serum-free media for 1 h prior to addition of *C*. *albicans*. Media was replaced with fresh serum free media containing 5 x10^5^ *C*. *albicans* cells and 50 μg/ml glucan phosphate, and cells co-incubated for 1 h. Attachment was scored in ImageJ. Data were analysed by Kruskal-Wallis test followed with a post-hoc Dunn’s multiple comparisons test at 95% confidence.

### Neutrophil isolation

Peripheral whole blood (18 ml) was taken from healthy volunteers and immediately laid under dual Percoll (GE Healthcare) density gradients of 1.098 and 1.079. Gradients were centrifuged at 150 x g for 8 min, followed by 1200 x g for 10 min. The neutrophil layer was removed to a fresh tube containing 3 volumes of red blood cell lysis buffer (0.83% NH_4_Cl, 0.1% KHCO_3_, 0.004% Na_2_EDTA.2H_2_O and 0.25% BSA) and gently agitated for 3 min to lyse contaminating red blood cells. Neutrophils were centrifuged at 400 x g for 6 min, and the resulting pellet washed twice in sterile endotoxin free PBS. Neutrophils were resuspended in serum free RPMI supplemented with 100 mM L-glutamine at a cell concentration of 1 x10^5^ cells/ml.

### Neutrophil phagocytosis assay

Two hundred microlitres of neutrophils (0.2 x10^5^ cells) were co-incubated with 1 x10^5^ *C*. *albicans* cells (MOI = 5) grown to mid-exponential phase in buffered YPD for 1 h. Non-phagocytosed *C*. *albicans* cells were removed by repeated washing with sterile PBS and cells fixed with 4% PFA for 15 min. Cells were washed once with PBS to remove PFA and cells were wet mounted and immediately imaged in duplicate. At least 300 neutrophils were counted for each condition per experiment. Data were analysed by one-way ANOVA followed by post-hoc Tukey test at 95% confidence.

### Stimulation of peripheral blood mononuclear cells

PBMCs were isolated as described above, except the monocyte layer was extracted from the Percoll gradient. NGY152 was grown to exponential phase in YPD buffered at the appropriate pH, washed in PBS and resuspended to the desired cell concentration. Yeast cells were fixed with 4% PFA for 30 min washed three times with PBS and 0.5 x10^5^ *C*. *albicans* cells were added to 2.5 x10^5^ PBMCs (MOI = 0.5) in a final volume of 200 μl. Samples were incubated at 37°C, 5% CO_2_ for 24 h. Samples were centrifuged and supernatant transferred into a fresh 96-welled plate and stored at -20°C. Extracellular cytokines were measured using commercially available ELISA kits (R&D) according to the manufacturer’s recommendations. Data were analysed by two-way ANOVA, followed by post-hoc test at 95% confidence level.

### Neutrophil recruitment assay

*C*. *albicans* NGY152 yeast cells were inoculated in YPD and grown overnight at 37°C, 200 rpm. Cells were sub-cultured into YPD buffered at the appropriate pH to an OD_600_ of 0.1 and grown until mid-exponential phase at 37°C, 200 rpm. Cells were harvested, washed three times in sterile, endotoxin free saline and resuspended in saline to 1 x10^8^ cells/ml for subsequent inoculation into mice. Cell counts were verified by plating for CFUs.

BALB/c female mice (n = 5/group, 8–12 weeks old, Envigo, UK) were injected intraperitoneally with the prepared inocula at 10^7^ *C*. *albicans* in 100 μL sterile saline. Group size was determined from previous experiments as the minimum number of mice needed to detect statistical significance (p<0.05) with 90% power. Mice were randomly assigned to groups by an investigator not involved in the analysis and the fungal inocula were randomly allocated to groups. Mice were housed in Individually Ventilated Cages (IVCs) and were provided with food and water *ad libitum*. Inocula were delivered in an unblinded fashion. After 4 h, mice were sacrificed by IV euthatal injection. Immune infiltrates were collected by peritoneal lavage [51]. Cells were stained to discriminate live/dead cells using a UV fixable live/dead dye (Thermo) and then fixed in 2% PFA. Cells were stained for CD45, CD11b, and Ly-6B.2 (7/4 clone) to differentiate lymphocytes, and F4/80 and Ly6G to differentiate neutrophils and macrophages. Using flow cytometry, 10,000 cells were analysed for each mouse. Cells were analysed on a BD Fortessa flow cytometer, with automatic compensation protocols. Statistical analyses were performed using Graph Pad Prism (v 7). Significance was determined using Welch’s t-test for unpaired data. Bars represent 95% CI. Variance between the groups for total cell counts was statistically different (F test to compare variances, p = 0.0354). All animal experiments were performed under UK Home Office project license PPL 70/902760/4135 granted to DMM in accordance with Home Office ethical guidelines. Work was performed by DMM and ERB.

## Supporting information

S1 Fig De-cloaking of the fungal cell wall in response to environmental pH is an active process. *C*. *albicans* cells were grown overnight in YPD. Cells were killed by fixing with 4% PFA, heat killing at 65°C for 2 h, treatment with 1 J UV light, or 100 mM thimerosal for 45 mins. Cells were washed and incubated in YPD buffered at pH2, 4 and 6 for 4 h. Cells were stained for **a)** chitin and **b)** β-glucan exposure. Data represent the mean and SEM from three biological repeats (* p < 0.05, ** p < 0.01, **** p <0.001).
(TIFF)

S2 Fig Release of the outer phosphomannan does not result in unmasking of inner cell wall components. *C*. *albicans* strains were grown to mid-log phase in YPD and YPD buffered at pH4, fixed with 4% PFA and carbohydrate exposure quantified by immunofluorescence. Fluorescence was quantified by FACS analysis of 10,000 events per strain, per condition, per repeat and is expressed as the fold-increase at pH4 relative to YPD. Data represent the mean ± SEM from three independent repeats.
(TIFF)

S3 Fig Unmasking of β-glucan in response to environmental pH is not mediated via conventional cell wall or pH sensing pathways. **a)** β-glucan unmasking in kinase mutants grown to mid-log phase in YPD buffered to pH4 as quantified by FACS analysis of immunofluorescent staining and repressed as fold change relative to YPD. Data represent the mean ± SEM from three independent experiments. **b)** β-glucan unmasking in *C*. *albicans* transcription factor mutants grown to mid-log phase in YPD buffered to pH4 as quantified by FACS analysis of immunofluorescent staining and repressed as fold change relative to YPD. Data represent the mean ± SEM from three independent experiments. **c)** β-glucan unmasking in Rim101 pathway mutants grown to mid-log phase in YPD buffered to pH4 as quantified by FACS analysis of immunofluorescent staining and repressed as fold change relative to YPD. Data represent the mean ± SEM from three independent experiments.
(TIFF)

S4 Fig The *rim101*Δ and *bcr1*Δ mutants display reduced phagocytosis. *C*. *albicans* strains were grown in YPD at the appropriate pH to mid-log phase, co-incubated with J774.1A macrophages at an MOI = 5 for 1 h and the **a)** phagocytosis index and **b)** association index determined. Data represent the mean ± SEM from three independent repeats. **c)** PBMCs were incubated with PFA fixed mid-log phase cells at an MOI of 0.5 for 24 h and TNFα secretion quantified by ELISA. Data represent the mean ± SEM from three donors in triplicate (* p < 0.05).
(TIFF)

S1 Table *C*. *albicans* strains used in this study. (DOCX)

S1 References Supplemental references. (DOCX)
