# Supplementary figures and images for: Adaptation of Candida albicans to environmental pH induces cell wall remodelling and enhances innate immune recognition
Source: PLoS Pathog. 2017 May 22;13(5):e1006403. doi: 10.1371/journal.ppat.1006403 (PMC5456412; doi:10.1371/journal.ppat.1006403)

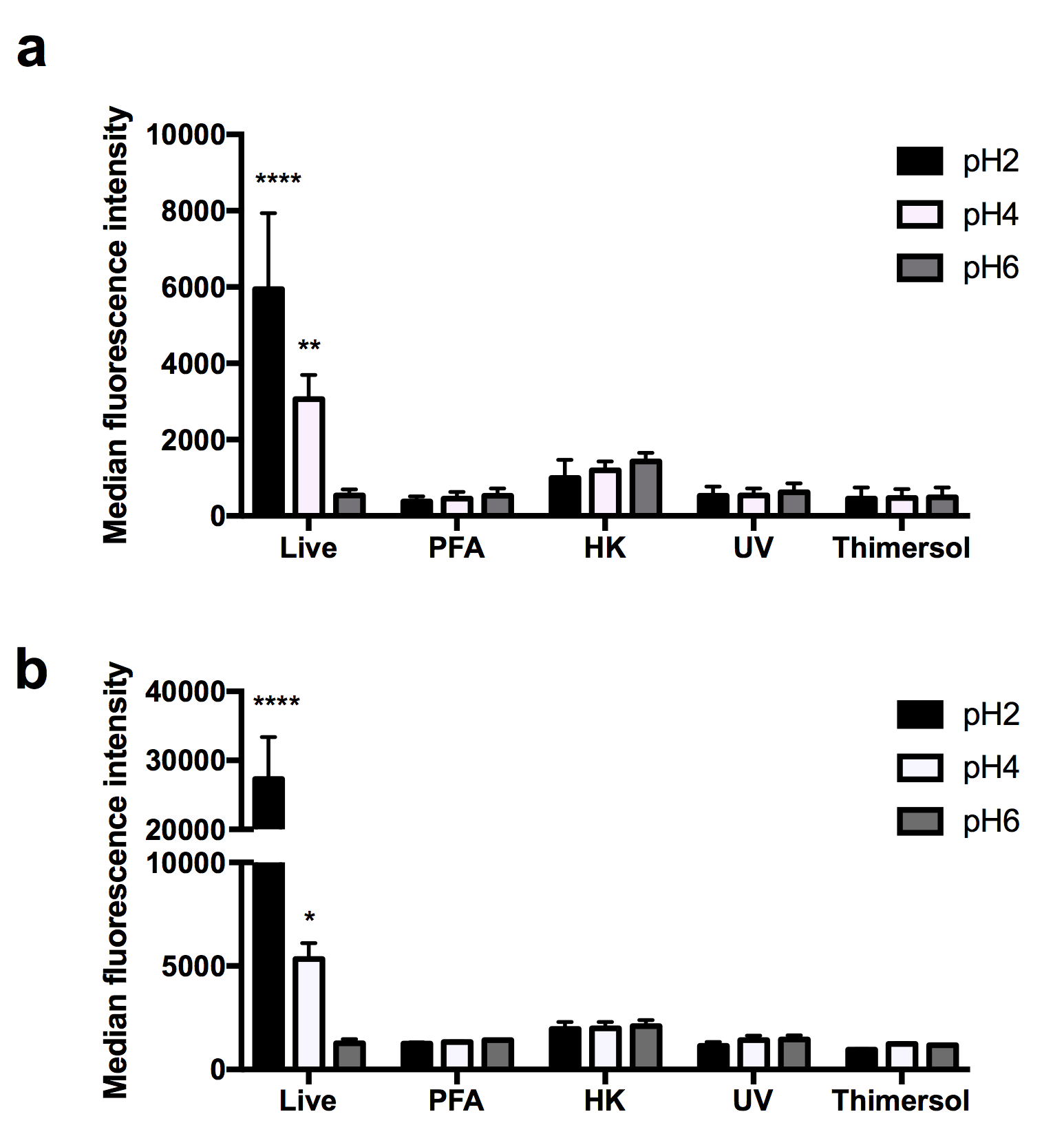

Supplement: S1 Fig — C. albicans cells were grown overnight in YPD. Cells were killed by fixing with 4% PFA, heat killing at 65°C for 2 h, treatment with 1 J UV light, or 100 mM thimerosal for 45 mins. Cells were washed and incubated in YPD buffered at pH2, 4 and 6 for 4 h. Cells were stained for a) chitin and b) β-glucan exposure. Data represent the mean and SEM from three biological repeats (* p < 0.05, ** p < 0.01, **** p <0.001). (TIFF) [file ppat.1006403.s001.tiff]

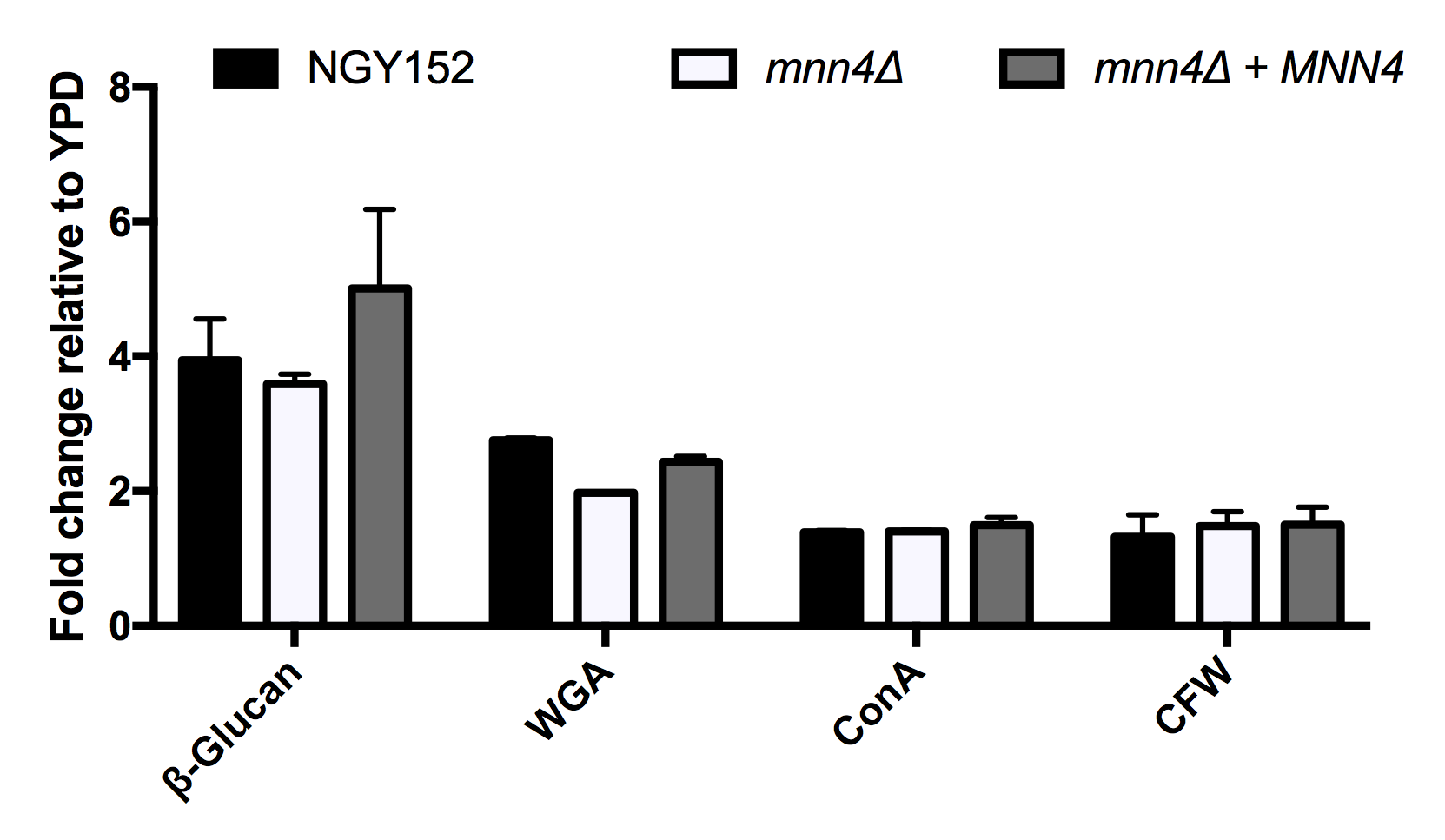

Supplement: S2 Fig — C. albicans strains were grown to mid-log phase in YPD and YPD buffered at pH4, fixed with 4% PFA and carbohydrate exposure quantified by immunofluorescence. Fluorescence was quantified by FACS analysis of 10,000 events per strain, per condition, per repeat and is expressed as the fold-increase at pH4 relative to YPD. Data represent the mean ± SEM from three independent repeats. (TIFF) [file ppat.1006403.s002.tiff]

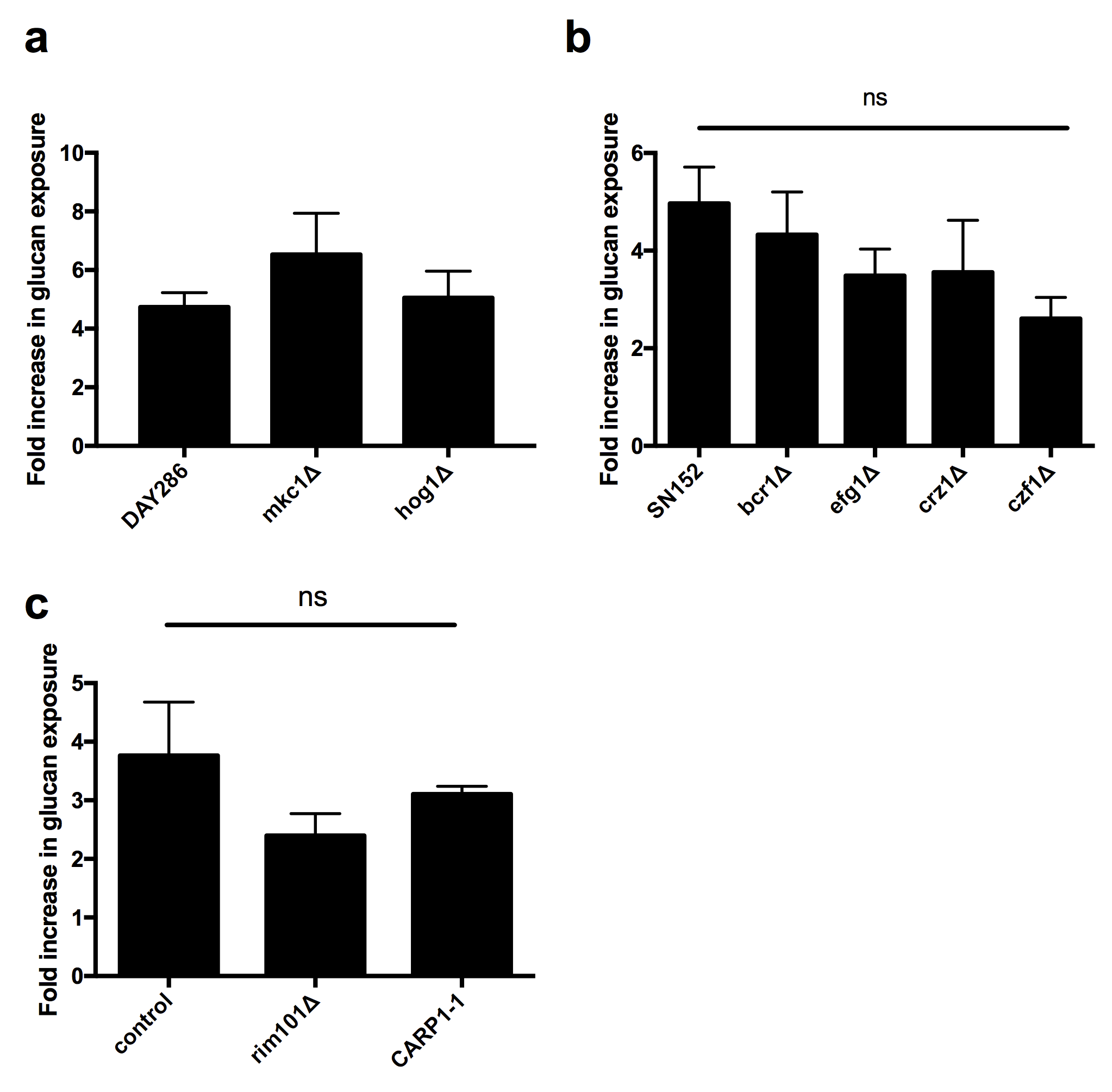

Supplement: S3 Fig — a) β-glucan unmasking in kinase mutants grown to mid-log phase in YPD buffered to pH4 as quantified by FACS analysis of immunofluorescent staining and repressed as fold change relative to YPD. Data represent the mean ± SEM from three independent experiments. b) β-glucan unmasking in C. albicans transcription factor mutants grown to mid-log phase in YPD buffered to pH4 as quantified by FACS analysis of immunofluorescent staining and repressed as fold change relative to YPD. Data represent the mean ± SEM from three independent experiments. c) β-glucan unmasking in Rim101 pathway mutants grown to mid-log phase in YPD buffered to pH4 as quantified by FACS analysis of immunofluorescent staining and repressed as fold change relative to YPD. Data represent the mean ± SEM from three independent experiments. (TIFF) [file ppat.1006403.s003.tiff]

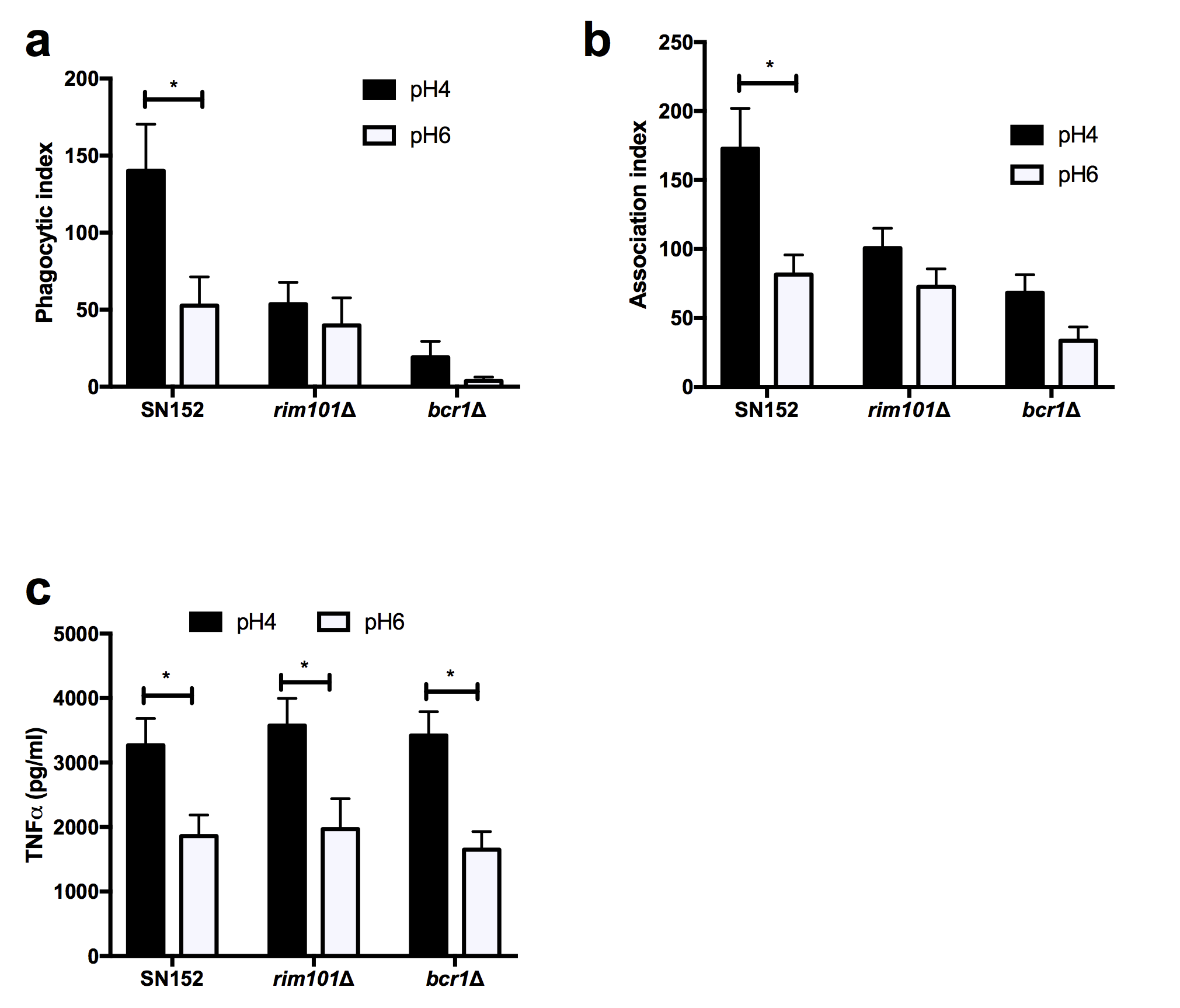

Supplement: S4 Fig — C. albicans strains were grown in YPD at the appropriate pH to mid-log phase, co-incubated with J774.1A macrophages at an MOI = 5 for 1 h and the a) phagocytosis index and b) association index determined. Data represent the mean ± SEM from three independent repeats. c) PBMCs were incubated with PFA fixed mid-log phase cells at an MOI of 0.5 for 24 h and TNFα secretion quantified by ELISA. Data represent the mean ± SEM from three donors in triplicate (* p < 0.05). (TIFF) [file ppat.1006403.s004.tiff]
