# Supplementary material for: Adaptation of Candida albicans to environmental pH induces cell wall remodelling and enhances innate immune recognition
Source: PLoS Pathog. 2017 May 22;13(5):e1006403. doi: 10.1371/journal.ppat.1006403 (PMC5456412; doi:10.1371/journal.ppat.1006403)
Supplement: S1 Table — (DOCX) [file ppat.1006403.s005.docx]

| **Strain** | **Species** | **Genotype** | **Reference** |
| --- | --- | --- | --- |
| SC5314 | *C. albicans* | Typed Strain | [1] |
| NGY152 | *C. albicans* | *λimm434/ura3*Δ::*λimm434 RPS1/rps1::URA3* | [2] |
| DAY286 | *C. albicans* | *λimm434/ura3*Δ::*λimm434 his1*Δ *hisG*/*his1*Δ::h*isG* *arg4*Δ::*hisG*-*ARG4*-*URA3*/*arg4*Δ::*hisG* | [3] |
| SN152 | *C. albicans* | *arg4Δ/arg4Δ leu2Δ/leu2Δ his1Δ/his1Δ URA3/ura3Δ:: λimm434 IRO1/iro1Δ:: λimm434* | [4] |
| CAF2-1 | *C. albicans* | URA3/ura3:: *λ*imm434 | [5] |
| CAY676 | *C. tropicalis* | Typed strain | ATCC |
| WU284 | *C. dublinensis* | Typed strain | [6] |
| AM16/0701 | *C. krusei* | Clinical isolate | From D. MacCallum, University of Aberdeen |
| CLIB214 | *C. paraosilosis* | Typed strain | [7] |
| ATCC 2001 | *C. glabrata* | Typed strain | ATCC |
| *rim101*Δ | *C. albicans* | *arg4Δ/arg4Δ leu2Δ/leu2Δ his1Δ/his1Δ URA3/ura3Δ:: λimm434 IRO1/iro1Δ:: λimm434 rim101::LEU/rim101::HIS1* | [8] |
| *mkc1*Δ | *C. albicans* | *λimm434/ura3*Δ::*λimm434*  *his1*Δ *hisG*/*his1*Δ::h*isG* *arg4*Δ::*hisG*-*ARG4*-*URA3*/*arg4*Δ::*hisG mkc1::URA3/mkc1::ARG3* | [9] |
| *rlm1*Δ | *C. albicans* | *arg4Δ/arg4Δ leu2Δ/leu2Δ his1Δ/his1Δ URA3/ura3Δ:: λimm434 IRO1/iro1Δ:: λimm434 rlm1::LEU/rlm1::HIS1* | [8] |
| *bcr1*Δ | *C. albicans* | *arg4Δ/arg4Δ leu2Δ/leu2Δ his1Δ/his1Δ URA3/ura3Δ::λimm434 IRO1/iro1Δ:: λimm434 bcr1::LEU/bcr1::HIS1* | [8] |
| *crz1*Δ | *C. albicans* | *arg4Δ/arg4Δ leu2Δ/leu2Δ his1Δ/his1Δ URA3/ura3Δ:: λimm434 IRO1/iro1Δ:: λimm434 crz1::LEU/crz1::HIS1* | [8] |
| *efg1*Δ | *C. albicans* | *arg4Δ/arg4Δ leu2Δ/leu2Δ his1Δ/his1Δ URA3/ura3Δ:: λimm434 IRO1/iro1Δ:: λimm434 efg1::LEU/efg1::HIS1* | [8] |
| *czf1*Δ | *C. albicans* | *arg4Δ/arg4Δ leu2Δ/leu2Δ his1Δ/his1Δ URA3/ura3Δ:: λimm434 IRO1/iro1Δ:: λimm434 czf1::LEU/czf1::HIS1* | [8] |
| *cht1*Δ | *C. albicans* | *λimm434/ura3*Δ::*λimm434 hisG*/*his1*Δ::h*isG* *arg4*Δ::*hisG*-*ARG4*-*URA3*/*arg4*Δ::*hisG cht1::URA3/cht1::ARG3* | [10] |
| *cht2*Δ | *C. albicans* | *λimm434/ura3*Δ::*λimm434 hisG*/*his1*Δ::h*isG* *arg4*Δ::*hisG*-*ARG4*-*URA3*/*arg4*Δ::*hisG cht2::URA3/cht2::ARG3* | [10] |
| *cht3*Δ | *C. albicans* | *λimm434/ura3*Δ::*λimm434 hisG*/*his1*Δ::h*isG* *arg4*Δ::*hisG*-*ARG4*-*URA3*/*arg4*Δ::*hisG cht3::URA3/cht3::ARG3* | [10] |
| CARP1-1 | *C. albicans* | *prr1Δ*::hisG/*prr1Δ*::*hisG*-*URA3*-*hisG*  *ura3Δ*: *λimm434*/ura3*Δ*:: *λimm434* *RIM101^1697A^* | [11] |
| *hog1*Δ | *C. albicans* | *λimm434/ura3*Δ::*λimm434 hisG*/*his1*Δ::*hisG* *arg4*Δ::*hisG*-*ARG4*-*URA3*/*arg4*Δ::*hisG hog1::URA3/hog1::ARG3* | (51) |
| hAHGI (GFP-Hog1) | *C. albicans* | *λimm434/ura3*Δ::*λimm434* hog1 : : hisG/hog1 : : hisG ACT1p-HOG1-GFP : : leu2/LEU2 | [12] |
| 302 | *C. albicans* | Clinical isolate from urine | National Institute for Health Research Surgical Reconstruction and Microbiology Research Centre, Queen Elizabeth Hospital, Birmingham, United Kingdom |
| 300 | *C. albicans* | Clinical isolate from blood |  |
| 304 | *C. albicans* | Clinical isolate from sputum |  |
| 316 | *C. albicans* | Clinical isolate from burn wound |  |
| 317 | *C. albicans* | Clinical isolate from wound drain |  |
| 320 | *C. albicans* | Clinical isolate from wound |  |
| CDH14 | *C. albicans* | *ura3*Δ::*λimm434/ura3*Δ::*λimm434 mnn4*Δ::*hisG/mnn4*Δ::*hisG, RP10*::[*MNN4-URA3*-RP10] | [13] |
| CDH15 | *C. albicans* | *ura3*Δ::*λimm434/ura3*Δ::*λimm434 mnn4*Δ*::hisG/mnn4*Δ::*hisG*, *RP10::URA3* | [13] |
| *she3*Δ | *C. albicans* | she3Δ/she3Δ; ura3Δ/ura3Δ | [14] |
