# Supplementary material for: Adaptation of Candida albicans to environmental pH induces cell wall remodelling and enhances innate immune recognition
Source: PLoS Pathog. 2017 May 22;13(5):e1006403. doi: 10.1371/journal.ppat.1006403 (PMC5456412; doi:10.1371/journal.ppat.1006403)
Supplement: S1 References — (DOCX) [file ppat.1006403.s006.docx]

S1 references

1. Gillum AM, Tsay EYH, Kirsch DR. Isolation of the *Candida albicans* gene for orotidine-5′-phosphate decarboxylase by complementation of *S. cerevisiae* *ura3* and *E. coli* pyrF mutations. Mol and Gen Genet. 1984;198(1):179-82.

2. Brand A, MacCallum DM, Brown AJP, Gow NAR, Odds FC. Ectopic Expression of *URA3* Can Influence the Virulence Phenotypes and Proteome of *Candida albicans* but Can Be Overcome by Targeted Reintegration of *URA3* at the *RPS10* Locus. Eukaryotic Cell. 2004;3(4):900-9. doi: 10.1128/ec.3.4.900-909.2004.

3. Davis DA, Bruno VM, Loza L, Filler SG, Mitchell AP. *Candida albicans* Mds3p, a conserved regulator of pH responses and virulence identified through insertional mutagenesis. Genetics. 2002;162(4):1573-81. PubMed PMID: PMC1462392.

4. Noble SM, Johnson AD. Strains and Strategies for Large-Scale Gene Deletion Studies of the Diploid Human Fungal Pathogen *Candida albicans*. Eukaryotic Cell. 2005;4(2):298-309. doi: 10.1128/EC.4.2.298-309.2005. PubMed PMID: PMC549318.

5. Fonzi WA, Irwin MY. Isogenic Strain Construction and Gene Mapping in *Candida albicans*. Genetics. 1993;134(3):717-28. PubMed PMID: PMC1205510.

6. Staib P, Moran G, Sullivan D, Coleman D, Morschhäuser J. sogenic strain construction and gene targeting in *Candida dubliniensis.* J Bacteriol 2001;183(9):2859-65.

7. Rossignol T, Ding C, Guida A, d'Enfert C, Higgins DG, Butler G. Correlation between Biofilm Formation and the Hypoxic Response in *Candida parapsilosis*. Eukaryotic Cell. 2009;8(4):550-9. doi: 10.1128/EC.00350-08. PubMed PMID: PMC2669199.

8. Homann OR, Dea J, Noble SM, Johnson AD. A Phenotypic Profile of the *Candida albicans* Regulatory Network. PLoS Genet. 2009;5(12):e1000783. doi: 10.1371/journal.pgen.1000783.

9. Blankenship JR, Fanning S, Hamaker JJ, Mitchell AP. An Extensive Circuitry for Cell Wall Regulation in *Candida albicans*. PLoS Pathog. 2010;6(2):e1000752. doi: 10.1371/journal.ppat.1000752.

10. Norice CT, Smith FJ, Solis N, Filler SG, Mitchell AP. Requirement for *Candida albicans* Sun41 in Biofilm Formation and Virulence. Eukaryotic Cell. 2007;6(11):2046-55. doi: 10.1128/EC.00314-07. PubMed PMID: PMC2168420.

11. Porta A, Wang Z, Ramon A, Muhlschlegel FA, Fonzi WA. Spontaneous second-site suppressors of the filamentation defect of *prr1*Δ mutants define a critical domain of Rim101p in *Candida albicans*. Mol Genet Genomics 2001;266:624-31.

12. Arana DM, Nombela C, Alonso-Monge R, Pla J. The Pbs2 MAP kinase kinase is essential for the oxidative-stress response in the fungal pathogen *Candida albicans*. Microbiology. 2005;151(4):1033-49. doi: doi:10.1099/mic.0.27723-0.

13. Hobson RP, Munro CA, Bates S, MacCallum DM, Cutler JE, Heinsbroek SEM, et al. Loss of cell wall mannosylphosphate in *Candida albicans* does not influence macrophage recognition. Journal of Biological Chemistry. 2004;279(38):39628-35. doi: 10.1074/jbc.M405003200.

14. Elson SL, Noble SM, Solis NV, Filler SG, Johnson AD. An RNA Transport System in *Candida albicans* Regulates Hyphal Morphology and Invasive Growth. PLOS Genetics. 2009;5(9):e1000664. doi: 10.1371/journal.pgen.1000664.
